# RNA Transcription and Splicing Errors as a Source of Cancer Frameshift Neoantigens for Vaccines

**DOI:** 10.1038/s41598-019-50738-4

**Published:** 2019-10-02

**Authors:** Luhui Shen, Jian Zhang, HoJoon Lee, Milene Tavares Batista, Stephen Albert Johnston

**Affiliations:** 1 The Biodesign Institute Center for Innovations in Medicine, Arizona State University, Tempe, AZ USA; 2 Present Address: Stanford University, Stanford, CA USA

**Keywords:** Immunosurveillance, Peptide vaccines

## Abstract

The success of checkpoint inhibitors in cancer therapy is largely attributed to activating the patient’s immune response to their tumor’s neoantigens arising from DNA mutations. This realization has motivated the interest in personal cancer vaccines based on sequencing the patient’s tumor DNA to discover neoantigens. Here we propose an additional, unrecognized source of tumor neoantigens. We show that errors in transcription of microsatellites (MS) and mis-splicing of exons create highly immunogenic frameshift (FS) neoantigens in tumors. The sequence of these FS neoantigens are predictable, allowing creation of a peptide array representing all possible neoantigen FS peptides. This array can be used to detect the antibody response in a patient to the FS peptides. A survey of 5 types of cancers reveals peptides that are personally reactive for each patient. This source of neoantigens and the method to discover them may be useful in developing cancer vaccines.

## Introduction

Checkpoint inhibitor immunotherapeutics are revolutionizing cancer therapy. However, even in the most responsive cancers a substantial portion (50–80%) of the patients have poor to no positive response^1–5^. A surprising finding in the analysis of these patients was that one of the best correlates of response has been the total number of neoantigens in the tumor^6–8^. This is also the case for patients with high microsatellite instability (MSI) where the production of FS neoantigens drives the effective anti-tumor immune responses^9–11^. The realization of the immunological importance of these DNA mutations has spawned the effort to develop personal vaccines^12^. As promising as early studies are of these vaccines, a major problem is that the majority of tumors will not have enough neoantigen-generating mutations to sustain development of a personal vaccine^13–15^. For example, melanoma tumors have a high mutational level with an average of 200 neoepitope mutations. This provides a large number to algorithmically screen for optimal antigenic presentation. In recent reports of two Phase I clinical trials of personal melanoma vaccines, starting with 90~2,000 personal neoantigens, 10 or 20 were identified for the vaccine^16,17^. However, in glioblastoma multiforme (GBM) only 3.5% patients had a high tumor mutation load, and further analysis showed that only a very small subset of GBM patients would potentially benefit from checkpoint blockade treatment^18^. This is also consistent with a lack of response in GBM patients to checkpoint inhibitors^19^. Massive genomic sequencing results indicated that GBM, ovarian cancer, breast adenocarcinoma and many other cancer types had very low number non-synonymous mutations, which will make these cancers difficult targets for personalized cancer vaccines^14,20^.

To solve this problem, we have investigated an alternative source of neoantigens which could possibly expand the scope of the application and efficacy of the neoantigen based cancer vaccines. In the process of becoming a tumor, not only does the DNA mutation rate increase with faster cell divisions, but also there is a disruption of basic cellular functions, including RNA transcription, splicing and the quality control system on peptides^21^. The disrupted RNA processes increase the FS transcripts generated by RNA splicing errors and the insertions and deletions (INDELs) of MSs^22^. Both of these processes, combined with the disrupted quality control system in tumor cells, can lead to the production of FS peptides and exposure of the FS epitopes to the immune system. Here we examine the possibility that FS variants produced by errors in RNA processing could be a source of cancer neoantigens and a simple system to detect them.

## Materials and Methods

### Cell lines and tissues

HEK293, B16-F10 and 4T1 cell lines were purchased from ATCC in 2006. Upon receipt, cells were cultured for three passages in RPMI medium (ATCC) with 10% FBS, 100 U/mL penicillin, and 100 mg/mL streptomycin and stored in aliquots under liquid nitrogen. Cells were maintained at 37 °C under humidified 5% CO_2_, 95% air. Cells between 2 and 20 passages were used. Cell lines were not re-authenticated. Other cells lines are listed in Supplementary Table S2 and were cultured in ATCC-recommended media.

### Mice and mouse tumor models

BALB/c and C57BL/6 mice were from Charles River Laboratories or Jackson Laboratories. For the tumor challenge, 5 × 10^3^ 4T1 cells were injected in the mammary pad at the right flank of the mice, or 1 × 10^5^ B16F10 cells were injected subcutaneously in the right flank of the mice. Tumor volumes were measured and calculated by (Length^2^ × Width/2) daily after the size was larger than 1 mm^3^. Breeding pairs of BALB-neuT and FVB-neuN (FVB/N-Tg (MMTVneu) 202Mul) mice were obtained from Joseph Lustgarten, Mayo Clinic Arizona. Mice were monitored weekly for the tumor incidence after tumor size reached 1 mm^3^. All experiments were performed in accordance with protocols approved by the Institutional Animal Care and Use Committee of Arizona State University. Statistical significance of differences was analyzed by a Student t-test.

### EST analysis

To identify potential putative chimeric transcripts, that when translated would result in a frame-shifted neo-peptide, we used two publicly available datasets and applied an algorithm that was used to identify chimeric transcripts. Specifically, we used the sequences found within the Expressed Sequence Taq (EST) library and the Human RefSeq database^23^ from the National Center for Biotechnology Information (NCBI). Using the stand-alone BLAST program, we aligned all EST sequences to RefSeq. We picked ESTs that aligned with 50–85 base pairs and had 95% homology to RefSeqs that have been previously annotated by National Center Institute (NCI). We further filtered out our alignment data by eliminating the EST sequences that did not align to multiple RefSeqs or were aligned in the 3′-5′ orientation. Lastly, we also eliminated the sequences that aligned with non-coding sequence regions. The remaining EST sequences were then used to identify the chimeric transcripts. Only the ESTs that aligned to two or more distinct RefSeq in consecutive positions were considered to be potential candidates. To be defined as a coding chimeric transcript, the EST sequences had to be at least 100 bp long with sequence similarity greater than or equal to 95% to the RefSeq. Also, the junction points between the two genes had to occur within the coding sequence of the upstream gene and orientation of the upstream gene alignment had to be in the positive (5′-3′) orientation. To eliminate false calls, all potential chimeric EST sequences had to be either present in more than one cDNA library or supported by three or more independent EST sequences. In addition, chimeric transcripts were classified based on the relative position of two genes. Classification of types of chimeric transcript was based on relative position of two fusion genes on the chromosome. Specifically, genes found on different chromosomes resulted in inter-chromosomal fusion while genes found in same chromosome were intra-chromosomal or read-through chimeric transcripts. Read-through chimeric transcripts resulted from two neighboring genes on same strand, otherwise intra-chromosomal.

### PCR screen for EST FS candidates

The 50 Human Breast cancer cell lines were obtained from the American Type Culture Collection (ATCC) and were grown according to recommendations. Human breast cancer tissue specimens were acquired from Mayo Clinic, and were informed consent and approval by the Mayo Clinic Institutional Review Board. All specimens were coded and anonymized. All experiments were performed in accordance with the approval protocol. Total RNA was extracted from breast cancer cell lines and primary breast tissues using the TRIzol LS reagent (Life Technologies, Carlsbad, CA) following the manufacturers protocol. RNA integrity was determined by gel electrophoresis and concentration was determined by measuring absorbance at 260/280 on the Nano-drop (NanoDrop Products, Wilmington, DE). cDNA was prepared by using the SuperScriptTM III First-Strand Synthesis SuperMix (Life Technologies, Carlsbad, CA) that includes random hexamers and oligo dT’s following the manufacturer’s recommended protocol. cDNA integrity and quality were assessed by performing a β-actin control PCR. End Point PCR primers for each chimeric transcript were designed using Primer3^24^ so that the forward and reverse primers both bind 80 bp to 280 bp upstream/downstream from the junction point. End-point PCR reactions using approximately 25 ng of cDNA, reagents from (Life Technologies, Carlsbad, CA) and 35 cycles were performed using Mastercycler ep gradient S (Eppendorf, Hamburg, Germany). PCR products were analyzed on 1.5% agarose gels. PCR products were purified, and sequence confirmed by Applied Biosystems 3730 (Life Technologies, Carlsbad, CA) sequencing.

### End-point RT-PCR

cDNAs from human primary breast tumors and normal mammary glands were from BioChain (Newark, CA). Total RNA from other sources was extracted with TRIzol (Life Technologies, Carlsbad, CA). cDNA was synthesized from total RNA using the SuperScript III First-Stand Synthesis SuperMix (Life Technologies). The primer sequences used for end-point RT-PCR were synthesized by Life Technologies or Sigma. End-point RT-PCR reactions (25 μL) used the GoTaq PCR kit (Promega, Madison, WI) and the following conditions: 95 °C for 2 min; 35 cycles of 95 °C for 30 secs, 60 °C for 30 sec (annealing), and 72 °C for 10 to 30 sec (extension); and 72 °C for 5 min. Exceptions were that mouse SMC1A primers used an annealing temperature of 55 °C, and β-actin primers were done with 25 cycles and 30 sec of extension time. Sequence verification was performed on RT-PCR products in initial reactions and later during intermittent reactions. We using following primers (from 5′ to 3′) for the PCR: SEC62 DNA human forward: TGCCATACCTGTTTTTCCC; sequencing results of microsatellite62 human DNA reverse: AGTTATCTCAGGTAGGTGTTGC; sequencing results of microsatellite62 DNA dog forward: AAGGGAGTCTGTGGTTGA; sequencing results of microsatellite62 DNA dog reverse: CAAAGAGGGAAGAGAGTGG; sequencing results of microsatellite62 cDNA human forward: AAAGGAAAAGCTGAAAGTGGAA; sequencing results of microsatellite62 human cDNA reverse: GCAACAGCAAGGAGAAGAATAC; sequencing results of microsatellite62 cDNA dog forward: AAGGGAGTCTGTGGTTGA; sequencing results of microsatellite62 cDNA dog reverse: CAAAGAGGGAAGAGAGTGG; SMC1A mouse forward: CTGTCATGGGTTTCCTG; SMC1A mouse reverse: GAGCTGTCCTCTCCTTG; SMC1A human forward:CCTGAAACTGATTGAGATTGAG; SMC1A human reverse: TCTTCAGCCTTCACCATTTC; β-actin mouse forward: CCAACCGTGAAAAGATGACC; β-actin mouse reverse: TGCCAATAGTGATGACCTGG; β-actin human forward: CCAACCGCGAGAAGATGACC; β-actin human reverse: TGCCAATGGTGATGACCTGG; Rat Her-2 forward: ATCGGTGATGTCGGCGATAT; Rat Her-2 reverse: GTAACACAGGCAGATGTAGGA.

### Sec62 transfection and flow analysis

HEK293 cell line were purchased from ATCC and cultured with standard protocols. Lipofectamine 2000 Transfection Reagent (Thermo Fisher Scientific, MA) was used to transfect plasmids into cell lines for overnight. Cells were then prepared in FACS buffer and quantified with flow cytometry. The three open reading frames (ORFs) were assembled by PCR and inserted into pCMVi vector at EcoR I MCS site. Detailed sequences of three ORFs were included in Table S7.

### Gene expression

Gene expression was measured with the TaqMan Gene Expression Assay (Life Technologies) according to the manufacturer’s directions. The hSMC1A-specific labeled probe was 5′-CAATGGCTCTGGGTGCTGTGGAATC-3′. The unlabeled forward and reverse primers were 5′-GGGTCGACAGATTATCGGACC-3′ and 5′-GTCATACTCCTGCGCCAGCT-3, respectively. Results were normalized by human GAPDH.

### Human frameshift peptide array and samples

Microsatellite Frameshift antigens: human mRNA sequences were acquired from NCBI CCDS databases^25^. Microsatellite regions (homopolymers of 7 runs or more) were mapped to human coding genes, 2^nd^ and 3^rd^ reading frame peptide sequences after MS regions were predicted and stored in Microsatellite FS database, MS FS peptides 10 aa or longer were included in the human FS peptide array.

Mis-splicing Frameshift antigens: human mRNA sequences and exon coordinates were acquired from NCBI Refseq database^23^. 2^nd^ and 3^rd^ reading frame FS peptide sequences were predicted from the start of every exon. Then all the FS peptides were aligned against the human proteome, FS peptides with higher than 98% homology to wildtype proteome were removed. FS peptides 10 aa or longer were then included in the human FS peptide array.

A total number of 64 non-cancer control samples and 13 pancreatic stage 1 cancer samples, 85 late stage cancer samples from 5 cancer types were tested on the FS array, detailed information was summarized in Table S6. All samples were acquired from collaborators and were informed consent upon collection through the institute’s own IRB. All samples were anonymized before receipt at Arizona State University (ASU) via Institutional Review Board (IRB) protocol No. STUDY00003722, ‘Receipt of Deidentified Human Serum for Immunosignature Analysis’ and protocol No. 0912004625, ‘Profiling Biological Sera for Unique Antibody Signatures’. All experiments were performed in accordance with the approval protocol.

### 400K FS peptide array assay

Serum was diluted 1:100 in binding buffer (0.01 M Tris-HCl, pH 7.4, 1% alkali-soluble casein, 0.05% Tween-20) and 150 ul diluted samples were loaded into each compartment of the 12-plex array and incubated overnight at room temperature or 4 °C. After sample binding, the arrays were washed 3X in wash buffer (1 × TBS, 0.05% Tween-20), 10 min per wash. Primary sample binding was detected via Alexa Fluor® 647-conjugated goat anti-human IgG secondary antibody (Jackson ImmunoResearch # 109-605- 098). The secondary antibody was diluted 1:10,000 (final concentration 0.15 ng/µl) in secondary binding buffer (1x TBS, 1% alkali-soluble casein, 0.05% Tween-20). Arrays were incubated with secondary antibody for 3 h at room temperature, washed 3X in wash buffer (10 min per wash), 30 secs in reagent-grade water, and then dried by centrifuging at 690 RPM for 5 mins. All washes and centrifugations were done on a Little Dipper 650 C Microarray Processor (SciGene) with preset programs. Fluorescent signal of the secondary antibody was detected by scanning at 635 nm at 2 µm resolution and 15% gain, using an MS200 microarray scanner (Roche NimbleGen).

### Plasmids for genetic immunization

The DNA fragments encoding FS peptides were cloned as a C-terminal fusion into the genetic immunization vectors pCMVi-UB^26^ and pCMVi-LSrCOMPTT^27,28^ with the Bgl II and Hind III and mixed with 1:1 ratio as the vaccine antigen. Three adjuvants were encoded by genetic immunization vectors. The pCMVi-mGM-CSF vector expresses the adjuvant mouse granulocyte/macrophage colony-stimulating factor (mGM-CSF) under control of the human cytomegalovirus (CMV) promoter^27^. LTAB indicates immunization with 1:5 ratio by weight of two plasmids, pCMVi-LTA and pCMVi-LTB, expressing the heat-labile enterotoxins LTA and LTB from Escherichia coli. These plasmids express LTA and LTB as C terminal fusions to the secretion leader sequence from the human α1 antitrypsin gene^29^. Vectors pCMVi-UB, pCMVi-LSrCOMPTT, pCMVi-LTA (also called pCMVi-LS-LTA-R192G) and pCMVi-LTB are available from the PSI:Biology-Materials Repository DNASU (dnasu.org) at Arizona State University. Additional adjuvants were the class A CpG 2216 single-stranded oligodeoxynucleotide obtained from Sigma and alum from Pierce.

### Bullet preparation for the genetic immunization with gene gun

Bullets for biolistic genetic immunization used the gold micronanoplex approach and were prepared as described^30^ with the following changes. Two grams of 1-micron gold was used. Prior to addition of N-hydroxysuccinimide and N-(3-dimethylaminopropyl)-N′-ethylcarbodiimide hydrochloride, the gold was resuspended in 20 mL of a 0.1 M solution of 2-(N-morpholino) ethanesulfonic acid (MES), pH 6.0. DNA-gold micronanoplexes were prepared by combining, per bullet, 57 μL of cysteamine-gold solution with precipitated DNA (≤10 μg) that had been resuspended in ≤15 μL of water, and then vortexing for 10 min. To the DNA-cysteamine-gold was added 6 μL/bullet of a freshly made solution of PEI-micron gold (167 mg/mL in 0.1 M MES, pH 6, without NaCl). The pelleted micronanoplexes were washed with ethanol prior to resuspension in n-butanol (55 μL/bullet), followed by bullet formation under nitrogen gas.

### Immunization dosage and regime and tumor challenge

#### C57BL/6-B16-F10 mouse melanoma model

Six week old mice (n = 10 per group) received one genetic immunization with the Gene Gun in the pinna of the ear (4 shots/mouse) with 20 ng of antigen (SMC1A-1^^^4 and non-protective Cowpox viral antigen CPV 172^31^) in pCMVi vectors plus the adjuvants pCMVi-mGM-CSF (0.5 µg) and CpG 2216 (5 µg) for each shot. All of the mice were challenged with 1 × 10^5^ B16-F10 cells 4 weeks after the immunization.

#### BALB/c-4T1 mouse breast tumor model

For the three MS FS experiments, all mice (n = 10 per group) were genetically immunized in the ear by Gene Gun at 8 weeks of age (2 shots/mouse, 60 ng pooled antigens plus 0.25 μg LTAB and 2.5 μg CpG2216 as the adjuvant for each shot) and boosted twice (two days apart) in three weeks with 1 μg pooled antigens plus the same adjuvants dosage. All mice were boosted again in two weeks with 50 μg KLH conjugated MS FS peptides with 50 μg CpG 2216 and 50 ul alum in total 100 ul PBS. The negative groups were immunized with the empty vectors and KLH protein with the same dosage. All mice were challenged with 5 × 10^3^ 4T1 cells two weeks after the last immunization.

For the mSMC1A-1^^^4 experiment, all mice were (n = 10 per group) genetically immunized in the ear by Gene Gun at 8 weeks of age (2 shots/mouse, 1 μg antigen plus 0.25 μg LTAB and 2.5 μg CpG2216 as the adjuvant for each shot), and boosted in two weeks with KLH conjugated SMC1A-1^^^4 peptide plus 50 μg Poly:IC (Sigma) in 100 ul PBS. The same regime was repeated in two weeks. The negative groups were immunized with the empty vectors and KLH protein with the same dosage. All mice were challenged with 5 × 10^3^ 4T1 cells 4 weeks after the last immunization. The CD8 and CD4 T cell depletion started 2 weeks after the last immunization by i.p injection of 100μg antibody (anti CD8, clone 2.43; anti CD4, clone GK 1.5; BioXCell, West Lebanon, NH) every 3 days until the end of the experiment.

#### BALB-*neuT* mice

Mice were genetically immunized by Gene Gun at 4-6 weeks with 100 ng of antigen(s) in pCMVi vectors, boosted twice (3-4 days apart) at 9-10 weeks with 1 μg of the same antigen(s), and boosted once at 13-14 weeks with protein. Genetic immunizations included adjuvants LTAB (0.5 μg) and CpG 2216 (5 μg). Protein boosts were 50 μg of KLH conjugated FS peptides (SMC1A-1^^^4, n = 32; RBM FS, n = 22; SLAIN2 FS, n = 14 and pool of three FS neoantigens, n = 37). The protein boost included 50 μg CpG 2216 and 50 μl alum in 100 μl PBS as the adjuvant. The negative groups (n = 30) were immunized with the empty vectors and GST or KLH protein with the same adjuvants and dosage.

### ELISPOT

Peptides used in the ELISPOT assays were synthesized in-house. The Mouse IFN-γ ELISPOT Set (BD Biosciences) was used according to the manufacturer’s directions except that blocking was at 37 °C. 10^6^ fresh mouse splenocytes were added to each well, followed by co-culturing for 48 hr with 20 μg of peptide in a volume of 200 μl RPMI medium. The plate was scanned and spots were analyzed by the AID EliSpot Reader System (Autoimmun Diagnostika GmbH, Germany).

### Statistics analysis

The statistical calculation software used was GraphPad Prism 7 (GraphPad Software, San Diego, CA) and JMP Pro (SAS Institute, NC). The data presentation and the statistical tests for each experiment are indicated in the legend of the corresponding figures, as well as the samples size and p-values.

### Significance

Personal cancer vaccines are promising as a new therapeutic treatment. These vaccines are currently based on mutations in tumor DNA. We show that variants in RNA production create frameshift neoantigens that may be another source of neoantigens for personal vaccines. Because there are only ~220 K of these antigens a simple peptide array can be used for their detection.

## Results

### A model for the production of RNA-based frameshift variants

**Figure 1:**
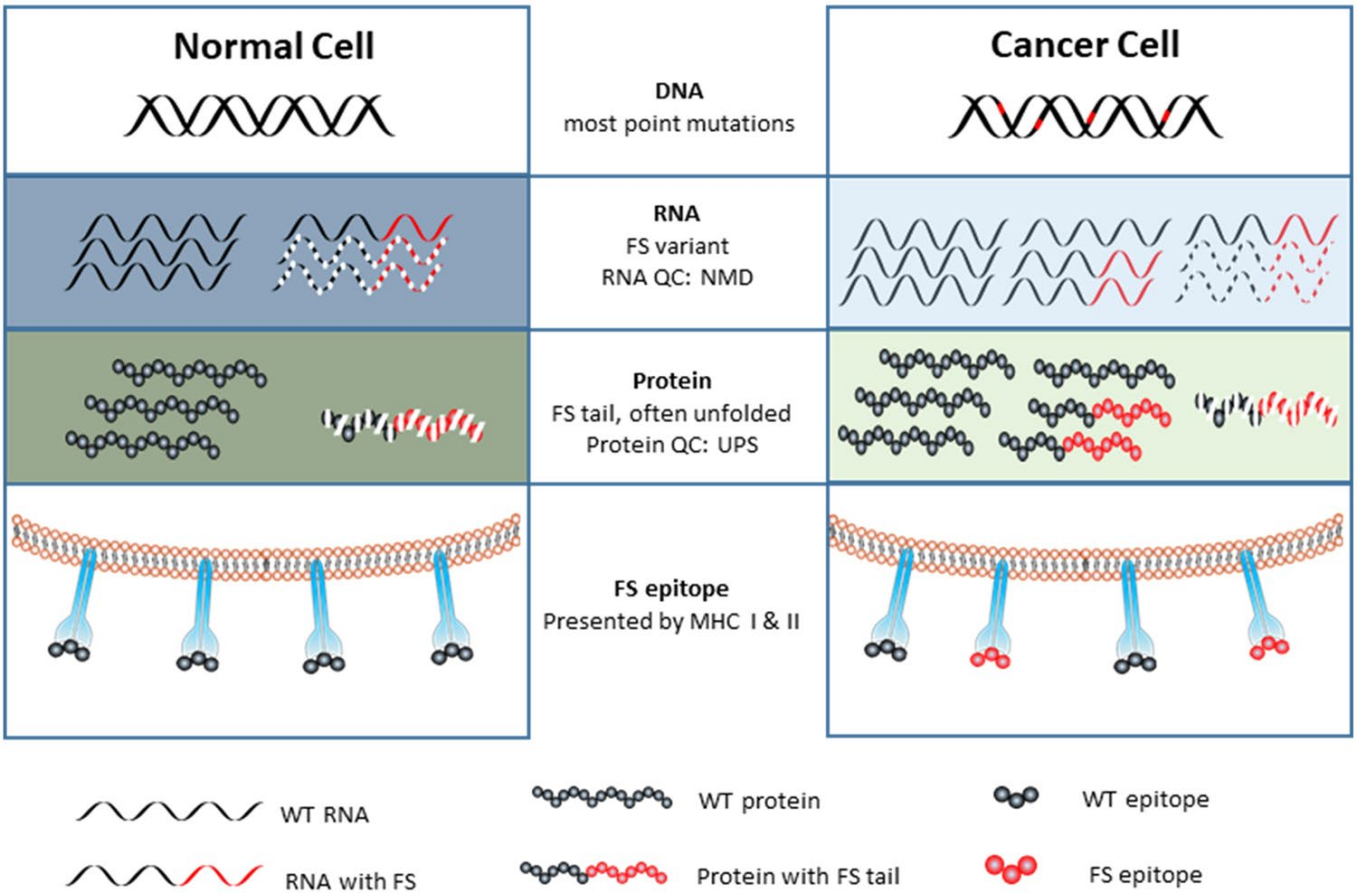
The model for RNA-based, frame-shift peptide production in tumor cells. Normal Cell: Errors in DNA replication are very rare and repaired. Transcription error rates are higher but also rare as are mis-splicing during intron excision. Additionally, the FS transcript with a premature termination may be degraded by Nonsense Mediated Decay (NMD). Aberrant proteins, including those with frameshifts are largely eliminated by the protein quality control system, Ubiquitin/Proteasome System (UPS). The net result is that very few frameshift peptides are presented on MHC I/II or escape the cell to be presented to the immune system. Cancer Cell: All levels of information transfer become more error prone. More errors are made in DNA replication, but only when cells divide. Most DNA mutations are point mutations and encode low or non-immunogenic epitopes. Global transcription is increased and is generally less accurate and even more so through MSs producing INDELs. Most transcribed genes with MSs in the coding region will have more FS transcripts. RNA splicing is also far less accurate, creating more FS transcripts from each out-of-frame splicing between exons from the same gene and different genes. The substantial increase of the FS transcripts from INDEls of MS and mis-splicing overwhelms the RNA quality control systems, such as NMD. Consequently, more truncated proteins with the FS peptide will be translated. These unfolded truncated proteins, combined with aberrant proteins from other mutations, overwhelms the protein quality control system, leading to more frameshift peptides being presented on MHC I/II and mis-secreted or released from the cancer cell which the immune system can respond to.

Here we propose that mistakes in RNA mis-splicing and transcription, particularly of INDELs of MSs in coding regions, in cancer cells may also be a source of neoantigens. The model that is the basis for this proposal and our efforts is presented in Fig. 1. As information flows from DNA to RNA to protein there is a general increase in error rates^22,32–35^. These errors include mis-splicing and INDELs of MSs. Both errors will produce a background level of FS transcripts, which encode truncated proteins with a FS peptide at the C-terminus. The level of the FS peptides in normal cells is managed by the quality control mechanisms, such as nonsense mediated decay^36^ and ER-associated degradation^37^, such that these FS peptides are not presented to the immune system. However, the initiation event of a potentially cancerous cell will destabilize basic cellular processes including transcription, RNA splicing and the quality control system^21,38–41^. These global errors can be augmented due to chromosomal instability^42^ or key, broadly effective mutations^43,44^. Consequently, the number of FS peptides produced, combining with other aberrant proteins, exceeds the disrupted quality control system, allowing FS peptides to be presented in MHC I/MHC II complexes or externally to dendritic cells. The level of FS production may be sufficient to be presented in MHC complexes but not induce a T-cell response. In most cases the aberrant cells are killed due to inherent dysfunction or by the immune system. Those escaping to become cancer cells could do so by decreasing MHC expression and/or establishing an immune suppressive environment. An important aspect of the model is that because of the global increase in the errors of transcription and splicing, the FS neoantigens will be constantly produced. Thus, in contrast to the commonly held view^45^, bystander FS neoantigens would be good immunological targets. The production of these variants is not dependent on DNA replication as is the case for DNA mutations nor are they heritable and subject to selection.

### Detection of FS transcripts

**Figure 2:**
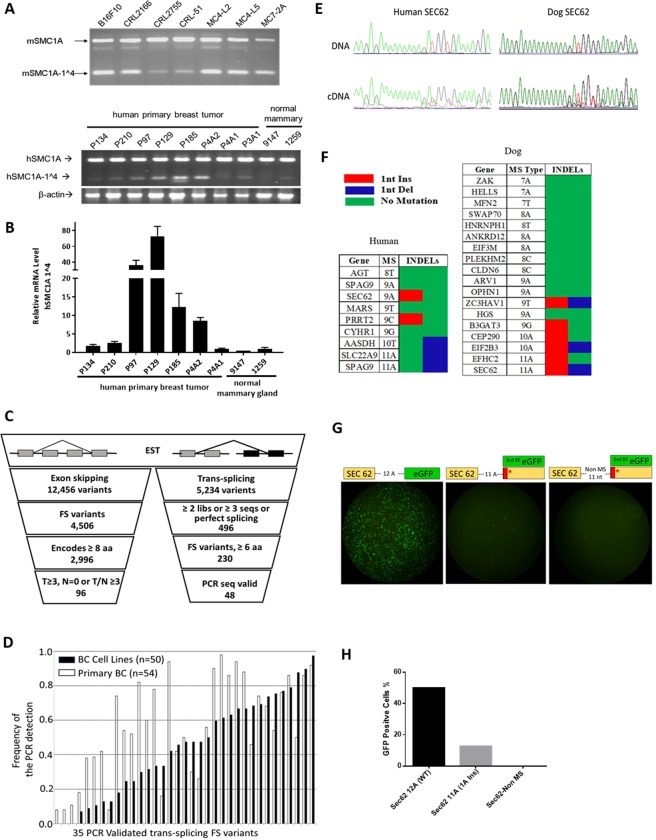
Detecting FS variants. (**A**) End-point RT-PCR analysis of the mSMC1A-1^^^4 in mouse tumor cell lines and human hSMC1A-1^^^4. (**B**) End-point RT-PCR analysis and RT-qPCR of the human hSMC1A-1^^^4 expression in human primary breast tumor tissues and normal mammary tissues. All values are normalized relative to the expression levels in sample 1259 (set as 1). Data are mean 2^−ΔΔC^ of triplicates with SD. (**C**) Analysis of the human EST database for FS variants by exon skipping and trans-splicing. (**D**) Analysis of the frequency of the expression of the 35 trans-splicing variants in 50 human breast cancer cell lines and 54 primary human breast tumors. (**E**) Example of a sequence trace of the MS region in SEC62 dog and human genes in paired DNA/cDNA samples. (**F**) Summary sequencing results of microsatellite candidates in human (4 breast cancer cell lines) and dog (primary dog tumor tissues). (**G**) *Ex vivo* analysis of the MS INDEL in transcription and translation of the MS INDEL variants. eGFP was fused to the 3^rd^ reading frame after 11 A MS of SEC62 or after 11 non-MS nucleotides. The eGFP directly fused to 12 A MS was the positive control. The three different plasmids were transfected individually into 293 T cells and GFP fluorescence was measured 24 hrs after transfection. (**H**) FACS analysis of the GFP positive cells.

This model makes several specific predictions. First, frequent FS variants in different cancers will be produced by errors in RNA splicing and transcription, not as DNA mutations. As an example of errors in mis-splicing, we find substantial levels of a FS transcript, SMC1A (exon 1 to exon 4), from the gene SMC1A in different mouse and human tumors (Figs 2A, S1A,E,F). The SMC1A1^^^4 encodes a 17 amino acids (aa) FS peptide (Fig. S1A). We did not detect the corresponding exon deletion in the DNA of mouse tumor cell lines, nor in the 12 TCGA cohorts (N = 4730) via Cancer Genomics Browser analysis (data not shown)^46^. Quantitative PCR demonstrates more expression of the SMC1A1^^^4 transcript in breast cancers than normal breast samples (Fig. 2B). To establish an estimate of the frequency of mis-splicing FS variants we sequenced 500 clones from a poly A-primed cDNA library of the mouse melanoma cell line, B16F10. We identified two FS variants SLAIN2_FS and ZDHHC17_FS, which skip exon 7 and 16 respectively (Fig. S1B,C). Interestingly, only SLAIN2_FS was detected in 4T1, a mouse breast tumor cell line (Fig. S1G). The same conserved FS variants were also detected in different human cancers (Fig. S1H). We noticed that while there were usually more (3-100-fold) frameshift transcripts in mis-splicing of these exons from tumor tissues or cancer cell lines, a low level of frameshift transcripts could be detected in some normal tissues (Figs 1B and S1I), which is consistent with the prediction of the model.

We expanded the analysis of RNA-generated FS variants by comparing NCBI tumor EST libraries to normal EST libraries. To simplify the analysis, we focused on FS variants caused by exon skipping or trans-splicing, i.e. splicing exons from different genes. A total of 12,456 exon skipping variants and 5,234 trans-splicing variants were found (Fig. 2C). 96 tumor associated FS variants from exon skipping passed the filters described in Fig. 2C, which also encode a FS peptide longer than 7 aa (Table S1). 230 FS trans-splicing variants that encode FS peptides longer than 6 aa were also identified. Primers were designed to screen 220 of these in different pools of cDNAs from 50 human breast cancer cell lines (Table S2) and 48 were successfully validated (Table S3). Two of these 48 FS variants, BCAS4-BCAS3 and MDS1-EVI1, have been described elsewhere^47,48^. 35 of these 48 FS variants were also found in 54 human primary breast tumors. The frequency of FS variants detected in tumor cell lines or tumor tissue is summarized in Fig. 2D. The expression frequency of these 48 variants range from 2% to 98% in tumor cell lines and primary tumors. Overall, a total of 27 out of 35 variants were expressed in over 50% of 50 tumor cell lines or 54 primary tumors. 12 of 35 variants tested were not detected in three normal tissues.

Another source of FS transcripts in tumors predicted by our model is INDELs in MSs generated in transcription. As an example, the microsatellite region in the Sec62 gene contains 9 and 11 repeats of Adenine in human and dog, respectively. The sequence of Sec62 and the corresponding INDEL frameshift peptides are shown in Fig. S2A. Human breast cancer cell lines and dog primary tumor tissues from 7 different cancer types were used for sequencing. No INDELs were detected at the genomic level. However, there was a significant level of one A insertion in the cDNA samples from the same tumor for both MSs (Fig. 2E). Two clones with one A insertion and one clone with one A deletion were found in sequencing 15 PCR clones from dog Sec62 cDNA. The INDEL rate was similar as estimated from the PCR sequence trace. 9 human MS candidates and 18 dog MS candidates were further sequenced in cDNA samples from cancer cell lines or primary tumor tissues. INDELs were frequently detected in MS candidates with repeat length of 9 or longer (Fig. 2F). This is consistent with large scale sequencing results in yeast^22^. The INDEL rate in transcription for MSs with repeat length of 7, 8 and 9 was very high compared to the genomic mutation rate but was not detected in the PCR sequencing trace due to low sensitivity of the assay. There is no evidence of INDELs in the MS in DNA in published reports except for Microsatellite Instability-High cancer patients with a defective mis-match repair system^15,49,50^.

To further validate the INDELs in the transcription and the translation of the FS peptide, we constructed three plasmids based on the dog Sec62 gene. One has the eGFP fused in the 3^rd^ reading frame to the MS region of 11 A in the dog Sec62 CDS. The eGFP protein will be correctly translated if there is one A insertion during the transcription. We replaced the 11 A with 11 nucleotides of non-MS sequence in another plasmid as the negative control, so there is no MS related INDEL in the transcription and no expression of eGFP. We also replaced the 11 A with 12 A as the positive control, so the eGFP is in the 1st reading frame and would be translated with the upstream dog Sec62 gene. (Fig. 2G). Plasmids were transfected into 293 T cells. 12.77% of the cells were GFP positive in the first construct which indicates this portion of the cells had 1 A insertions at the mRNA level and then successfully translated the FS protein. In contrast, none of cells were GFP positive in the negative control which indicates the MS region was crucial for INDELs (Fig. 2H). This experiment not only shows that the transcription could induce translatable FS variants with the INDELs in the MS region, but also indicates that FS peptides could be globally expressed in cancer cells with the defects in the quality control system.

### Detection of antibodies to FS peptides

The model also predicts that the increased expression of FS variants, combined with other aberrant proteins, would overwhelm the quality control system and could potentially elicit immune responses to these FS peptides. To test this, we designed an array of all possible predicted RNA-defined frameshift peptides, meeting specific qualifications that the tumor cell could produce from INDELs in coding MS and mis-splicing of exons.

**Figure 3:**
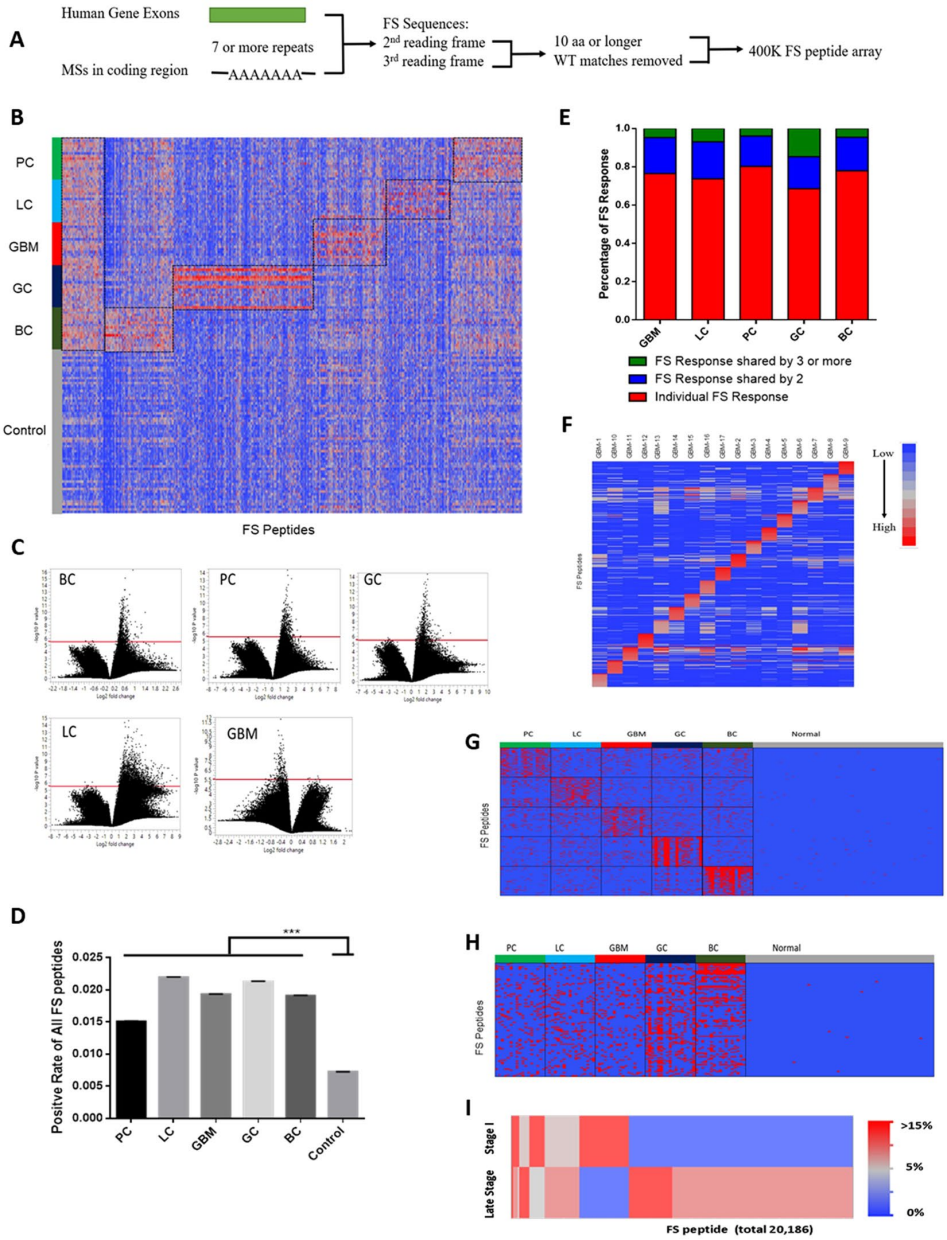
Detecting of antibody response against FS in cancer patients. (**A**) Design of human FS array with microsatellite FS peptides from all coding MS regions and predicted mis-splicing FS peptides from every exon of human genes. (**B**) Common reactivity and cancer-type reactivity against FS peptides were represented by ~7000 selected FS peptides. LC: lung cancer; BC: breast cancer; GBM: glioblastoma; GC: gastric cancer; and PC: pancreatic cancer (n = 17/each cancer type) and a set of non-cancer samples (n = 64), as control. (**C**) p-value and fold change volcano plot analysis of 5 cancer’s IgG reactivity on the 400 K FS array compared to normal. The red line represents the significant p-value cut-off = 1/392328 (the number of the array peptides). (**D**) Positive rate of all 400 K FS peptides in each cancer type, overall cancer and normal group (calculated by counting samples with higher reactivity than Average(Normal) + 6*SD (Normal)), error bar represents Mean ± SEM. (**E**) Distribution of personal anti-FS response and shared anti-FS response in all 5 cancer types. (**F**) Top 20 FS peptides for each GBM sample were selected for personal vaccines. (**G**) Components of cancer-type specific FS vaccines, top 100 FS peptides for each cancer type were selected with highest positive rate in corresponding cancer type. Red: positive sample, blue: negative sample. (**H**) Components of a general FS vaccine, top 100 FS peptides were selected with highest positive rate in cancer group. Red: positive sample, blue: negative sample. (**I**) Heatmap of the positive rate distribution of the FS peptides in Stage I and late stages pancreatic cancer.

There are over 8000 MS in the coding region of the human genome that are runs of 7 or more repeats of homopolymers. The majority of MS regions meeting our criteria are A runs and the number of MS candidates decreases exponentially as the repeat length or frameshift peptide length criteria increases (Fig. S2). Each MS could generate 2 predictable FS peptides depending on whether there was an insertion or deletion. In addition, there are ~200,000 possible FS peptides that could be generated by mis-splicing of exons in the human genome, such as the examples of mis-splicing FSs shown in Fig. S2B. Similar to MS FSs, the number of mis-splicing FSs decreases exponentially as the FS peptide length requirement increases. Most of mis-splicing FSs are generated from the first 10 exons of human genes (Fig. S2). We applied the restriction of the peptide being longer than 10 amino acids for both sources of FS. By these criteria there are over 220,000 possible FS antigens. We divided each FS antigen that was longer than 15aa into 15 aa, non-overlapping peptides. This produced a total of ~400,000 peptides. We also excluded peptides that share more than 10 aa identical sequences with any human reference proteins. Finally, we designed each FS array to contain a total of 392,318 FS peptides (Fig. 3A).

NimbleGen (Roche, Madison, WI) synthesized the FS peptide array, processed the array assay and summarized the IgG signals of each array with their standard protocol^51^. We analyzed the specific IgG reactivities to these FSPs in 64 non-cancer control samples and a total of 85 cancers from 5 different late stage cancer types with 17 samples each (LC: lung cancer, BC: breast cancer, GBM: glioblastoma, GC: gastric cancer, PC: pancreatic cancer) and 12 stage I pancreatic cancer samples.

Each array was normalized to its median florescence for analysis. Three patterns of FS feature reactivity that were higher in cancer than non-cancer were found: common reactivity against FS peptides across all 5 cancer types; cancer type specific reactivity and personal reactivity. Reactivity against ~7000 selected peptides are shown in Fig. 3B. Common reactivity and cancer type reactivity in 5 cancer types were marked with black squares. Non-cancer control samples had very low, sporadic reactivity in these FS peptides.

Total reactivity on the 400 K arrays was evaluated in the 5 cancer types and non-cancer samples with two methods. The first method compares the number of significant peptides in the cancer and control samples using fold change and p-values. By this method, BC, GC, PC and LC cancer samples had significantly more FS peptides compared to control samples which met the fold change and p-value criteria described in Fig. 3C. The exception is GBM where the reactivity in the controls was higher than the GBM samples. The second method used a scoring method for each FS peptide. A peptide is scored as positive (red) if it is higher than six times the standard deviation (6 SD) from the mean value of non-cancers for the peptide. All 5 cancer types had more positive FS peptides than the non-cancer controls (p-value < 0.0001, Fig. 3D).

The analysis of individual cancer samples within the same cancer type using the scoring method showed that there were three patterns of reactivity. Most of the positive FS peptides (69~80%) were personal for that individual. However, 16~19% of the positive peptides were shared between two samples in that cancer type, with 1.5~6.9% shared between 3 or more. The distribution of these classes is shown in Fig. 3E. Gastric cancer samples had the highest shared FS response (6.9% were shared in 3 or more). This is consistent with the very high correlation coefficients in several gastric cancer samples (Fig. S3G). Hierarchical clustering results of all positive FS peptides in the 5 cancer types are shown in Fig. S3.

Our model predicts that a FS peptide with high antibody reactivity is highly immunogenic and/or highly expressed in the tumor cells. These FS peptides could be cancer vaccine candidates. Analysis of the distribution of positive peptides allows the formulation 3 types of potential vaccines. One type is a personal vaccine. As an example, we show the personal vaccines for the 17 GBM patients. Each patient had ~5800 positive FS peptides using the 6 SD cut-off criterion and ~4500 positive FS peptides being unique for that patient.(Fig. S3B). We then applied a filter for highest binding signals to choose the 20 top peptides for each patient. These are depicted in Fig. 3F. This same system was applied to each of the other 4 cancer types with similar results (data not shown). It is noteworthy that even though GBM has been found to have a low DNA mutation rate^14^, there appear to be an abundance of reactive RNA variant FS peptide for which to create a vaccine.

As noted in Fig. 3B, there were also peptides that were commonly reactive in a cancer type. Based on this analysis a set of peptides could be chosen to optimize the number in common for a particular cancer. This is depicted in Fig. 3G for the 5 tumor types. The top 100 peptides based on the maximum coverage for the particular cancer type were chosen. We refer to these vaccine compositions as “focused” vaccines, as it is clear from the Fig. 3G that many of the peptides optimal for a particular cancer are shared across other cancer types.

Finally, we determined if there were FS peptides that were common across all 5 cancer types that met the p-value and frequency requirements. In Fig. 3H we present the best 100 candidate FS peptides for a pan-cancer (at least for the 5 considered) vaccine. It has been found that there are extremely few recurrent mutations in the DNA of certain tumors types^49^ and with low chance of being immunogenic. In contrast common reactive FS variants can readily be identified.

All of the samples used for this analysis were from patients with late stage cancer. Cancer vaccines could also potentially be used for treatment of early stage cancers. We were interested in whether early and late stage cancer vaccines would require different components. We compared the 20,000 most reactive and recurrent peptides compared to non-cancer for both the late stage and stage 1 pancreatic cancer. As evident in Fig. 3I, most of the peptides did not overlap between the late and early stages of pancreatic cancer. This implies that an early and late stage vaccine would require distinct peptide compositions.

### FS peptides offer partial protection as vaccines

The data presented above shows that FS variants are present at the RNA level in tumors and that antibody responses to these FS peptides are present in cancer patients. However, the clinically relevant question is whether these FS variants can afford therapeutic value as vaccines. We have explored this question using mouse tumor models.

**Figure 4:**
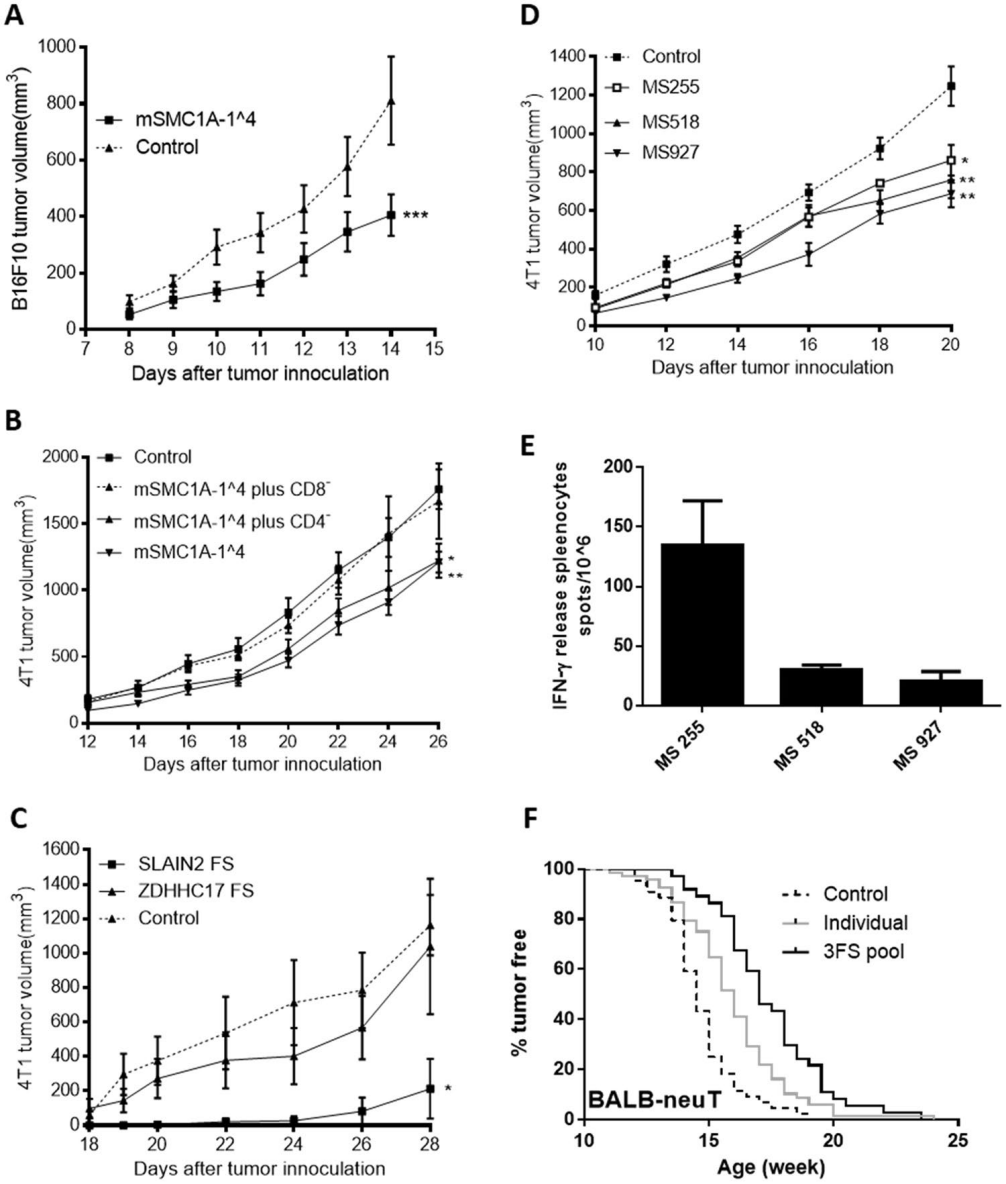
Protection of FS antigens as cancer vaccine candidates in different mouse tumor models. (**A**) Tumor growth curve of mSMC1A-1^^^4 immunization in the B16F10-C57BL6 tumor model compared to the control antigen, non-protective Cowpox viral antigen (CPV 172^31^) immunization. Mice (n = 10 per group) were genetically immunized at 8 weeks of age and challenged with 1 × 10^5^ B16-F10 cells 4 weeks later. (**B**) Tumor growth curve after mSMC1A-1^^^4 immunization in the 4T1-BALB/c tumor model. Mice (n = 10 per group) were prophylactically immunized and challenged 2.5 weeks after the last immunization by 5 × 10^3^ 4T1 cells. The CD8 and CD4 T cell depletion started 2 weeks after the last immunization. The control groups were genetically immunized with empty vectors and boosted with the KLH protein. (**C**) Tumor growth curve after FS neo-antigen immunization in the 4T1-BALB/c tumor model. Mice (n = 4 per group) were genetic immunized with SLAIN2 FS, ZDHHC17 FS and mock control three times in two week intervals and challenged 2 weeks after the last immunization by subcutaneous injection of 2 × 10^3^ 4T1 cells. (**D**) Three MS FS antigens were selected based on the best predicted H2D binding epitopes for BALB/C mice. The tumor growth curve is after three MS FS antigen immunizations in the 4T1-BALB/c tumor model. Mice (n = 10 per group) were prophylactically immunized with the different FS antigens or control antigen and challenged 2 weeks later with 5 × 10^3^ 4T1 cells. (**E**) ELISPOT analysis of the three MS FS neo-antigens immunizations. 3 mice were genetically immunized with a pool of the three MS FS neo-antigens and challenged with 5 × 10^3^ 4T1 cells. Splenocytes were collected 19 days after tumor challenge and a pool of three splenocytes were used in the assay. Error bars represent SD of triplicates. (**F**) Three FS antigens were selected and immunized individually or pooled in the BALB-NeuT mouse breast tumor model. A tumor free curve is presented of BALB-NeuT mice immunized with individual FS neo-antigens (SMC1A-1^^^4, n = 32; RBM FS, n = 22; and SLAIN2 FS, n = 14) (total n = 68), pool of these three FS neo-antigens (n = 37) and control group (total n = 44), including untreated (n = 14) and immunized with control antigens (n = 30). Control vs individual or 3 FS pool, p ≤ 0.0001; individual vs 3FS pool, p = 0.005. Error bars in all mouse growth curves represent SEM, *p < 0.05 and **p < 0.005 by two tailed t-test. Statistical analysis of the tumor free curve was with Mantal-Cox test.

We first determined if the SMC1A 1^^^4 FS peptide confers protection in the B16F10 mouse melanoma cancer model and/or the 4T1 mouse breast cancer model. This FS variant was shown to be common in both human and these mouse tumors (Figs 2A, and S1E). The FS peptide was encoded on a plasmid in a standard genetic immunization vector and introduced with a gene gun. 1 × 10^5^ B16F10 tumor cells were injected and the animals vaccinated 4 weeks later. The tumor volume was monitored and compared to control mice receiving a mock vaccination. As shown in Fig. 4A, the vaccine conferred significant retardation of tumor growth. The SMC1A 1^^^4 FS immunization also significantly retarded the 4T1 tumor growth in BALB/c mice (Fig. 4B). Depletion of CD8 or CD4 T-cells in the immunized mice indicates that this protection is CD8 T cell dependent (Fig. 4B).

We next tested whether the detection of FS variants in the RNA correlated with protection. The SLAIN2 and ZDHHC17 FSs had been identified in sequencing B16F10 cDNA. The SLAIN2 FS was present in the 4T1 mammary cancer cell line, but ZDHHC17 FS was not (Fig. S1G). When tested as gene vaccines in the mouse tumor injection model of 4T1, SLAIN2-FS conferred tumor retardation but ZDHHC17 did not (Fig. 4C).

The model (Fig. 1) implies that most transcribed genes with MSs in exons will produce FS peptides and these also may confer protection as vaccines. To test this prediction, we informatically chose 3 MS FSPs based on the peptide size and best predicted H2-D binding epitopes in the mouse MS FS database (Fig. 4D and Table S4). As predicted, each FS neoantigen vaccination significantly retarded the tumor growth compared to the control group (Fig. 4D). Each FS antigen also elicited specific IFN γ releasing splenocytes (Fig. 4E).

Our model also predicts that each tumor cell will present multiple FS neoantigens. These peptides could be presented at low levels as only a fraction of each RNA would be defective. Therefore, multiplexing neoantigens in a vaccine would be predicted to be more protective. To test this prediction, we tested three FS neoantigens individually and pooled together as vaccines in the BALB-NeuT transgenic mouse mammary cancer model. Each FS neoantigen-based vaccine individually showed similar protection by significantly delaying the tumor growth (Fig. S4). As predicted, the pooled neoantigen vaccine produced a significant additive increase in delaying tumor initiation and growth (Figs 4F and S4). This suggests that pooling multiple FS neoantigens will increase efficacy.

## Discussion

We present a model for how errors in transcription microsatellites and mis-splicing of exons could create frameshift neoantigens. We show examples in the RNA of tumors for both mis-splicing and of mis-transcription of an INDEL where the errors are present at the RNA but not DNA level. We also show that inspection of the NCBI EST library reveals other examples of FS variants. Using an array comprising all predicted FS peptides with specific qualifications, we demonstrate with human sera from patients with 5 different cancers have higher antibody reactivity than people without cancer. We show that 3 different patterns of high antibody reactive can be determined – pan-cancer, cancer-type focused and personal. Several examples are presented demonstrating that the FS variants offer at least partial protection in mouse models and that the protection is additive for each FS antigen.

If the model presented and the data supporting it are correct, it suggests that variants produced at the RNA level in tumor cells may be a good source of neoantigens for vaccines for several reasons. First, these FS variants produce neoantigens which are more likely to be immunogenic than neo-epitopes encoded by single nucleotide mutations^7^. Second, FS from MS INDELs would be particularly attractive sources. There are a limited number of possible variants (~8600 of homopolymers >= 7 bp), which encode about 7,000 FS peptides longer than 10 aa, thus reducing the search space for neoantigens. Third, because of the predictable number of candidates it should be possible to use a peptide array to screen for immune reactive neoantigens. This approach would be much simpler than sequencing tumor DNA obtained from a biopsy. Fourth, because any expressed gene has the potential to produce neoantigens, it may not be necessary to limit the vaccine to oncological driver genes. Finally, it should be difficult for the tumor cells to evolve away from the vaccine since these FSs are variants, not heritable mutations. Particularly if the FS antigen was produced in RNA from an essential gene, the tumor cells would need to restrict MHC presentation^17,52^ or create an immune suppressive environment^53^ to escape an immune response.

Elements of the model are supported by other published work. The immunological reactivity of FS neoantigens is the presumed basis of the effectiveness of PD-1 in most MSI-H cancers^54,55^. It also explains the responsiveness of renal cancer to CPI therapy - these cancers have low point mutation levels but high FS mutations^3,7,20^. It has also been shown that cancer cells have much higher mis-splicing rates than normal cells^39,41,56^. Recently, Andre *et al*.^56^ showed informatically that cancer cells could make neofusion sites by mis-splicing. However, their analysis did not include fusions that created FS peptides. Also, Alicia et.al.^57^ analyzed intron retention in tumor databases. This process can also create FS neoepitopes, though apparently much less frequently than mis-splicing of exons. The only aspects of the model not independently confirmed are 1) that the FS peptides potentially generated at the RNA level are made in tumors, 2) that the RNA-generated FSPs can generate immune responses, and 3) that these peptides can be protective against tumors. We believe the work presented here has supported these 3 remaining aspects of the model.

An important aspect of this source of neoantigens is that it may allow extending the personal vaccines to more patients and tumor types. Many tumors have relatively low numbers of DNA mutations and probably could not support constructing a vaccine^58^. We estimate from published mutational surveys of various tumors^59^, only 40% of patients could be treated with personal vaccines. However, our model predicts that the RNA FS variants would be produced in any cancer type, even if the DNA mutation level is low. We demonstrate this in GBM (Fig. 3B,G), which is a low mutation rate cancer^14,20^, but elicits similar overall immune response to FS peptides as other high mutation cancers.

The model also predicts that there may be recurrent FSs produced in different tumors. We demonstrated this at the RNA level for SMC1 FS in breast cancers (Fig. 2D). We also confirmed this to be the case by antibody reactivity using the FS arrays. We found 4641 FS peptides that were positive in 10% or more of all the samples across all five tumor types. We also found sets of FS peptides that had enriched activity in individual tumor types. A collection of a set of these peptides could potentially be used to constitute a general, therapeutic vaccine or one focused on a particular tumor or set of tumors. Such vaccines would have an advantage over a personal vaccine of being pre-made but would have fewer antigens in common with the tumor. Conceivably, pan-cancer peptides could be used to create a prophylactic cancer vaccine, as has been proposed for cancer associated antigens^60^. However, as we showed in comparing late and early stage pancreatic cancer profiles, a prophylactic vaccine from FS neoantigens would be best constituted from peptides reactive at early stages of cancer. We recently initiated a clinical trial in dogs of a prophylactic vaccine that is designed to be broadly protective (data not shown).

In Figs 3F and S3 we further refined the collection of reactive peptides to the personal level. Using GBM as an example, we can find a set of peptides that are personal for each patient. In the 17 patients analyzed there were 1316–8299 personal peptides which were reactive only in that individual. Approximately 70% of all cancer-specific reactivity on the arrays was personal. We present a set of 20 personal FS antigens for each GBM patient in Fig. 3F. The high antibody reactive indicates the high expression and/or high immunogenic of the FS antigen in the patient, with potential reactive CD4+ T cell response.

In Fig. 3G,H, we noted that people without cancer have sporadic antibody reactivity to some of the peptides. This has also been noted that healthy individuals have antibody and T-cell responses to tumor associated antigens^61,62^. This could be due to random background cross-reactive IgG antibodies unrelated to cancer. We have previously shown that monoclonal antibodies are capable of binding random sequence peptides with high affinity, even though the peptides do not contain a sequence resembling the cognate site^63^. Alternatively, this reactivity could be a manifestation of immune surveillance^64^ eliminating potential tumor cells. Any cell that produced and presented FS antigens, whether tumor or not, could be susceptible to this elimination, effectively a “bad cell” response.

The vaccines we tested did not produce complete protection by themselves in the models tested. However, it should be noted that both these models are very stringent and probably do not completely replicate natural tumors. We also think this is because there is low level production of each FS neoantigen, consistent with the additive effects of the FS peptides in vaccines (Figs 4F, S4). We have only occasionally been able to identify by mass spectrometry a FS peptide from MHC I elution of tumor cells, consistent with other reports^57^. The quantitative analysis of transcription errors reported by Gout *et al*. recently is consistent with this proposition^22,32^. However, this could also be due to the tumor cells deleting the antigen and evolving resistance, or that the T cell epitopes have low affinity, as is predicted for the mSMC1A FS peptide in the BALB/c mouse strain. Neoantigens produced by mutations in the DNA will produce 50–100% variant RNA and therefore potentially more presented antigen than would be expected for RNA based neoantigens. We have not detected pre-existing T-cell responses in our mouse tumor models, even though vaccination with the FS is at least partially protective. We propose that the level of RNA-error-based FS production in the tumor is generally not enough to elicit a T-cell response, but is enough to elicit T-cells elicited by a vaccine to kill the tumor cell. This is consistent with analysis of three clinical trials of personal vaccines^16,17,65^, where most of the antigens which produced a T-cell response had no pre-exiting T-cell response detectable. Recently, we have shown complete protection in the 4T1 model using pools of 10 selected FS antigens with both personal and cancer-type specific vaccines (MTB, LS and SAJ, data not shown).

The arrays detect antibody responses to FS peptides. B-cell responses are not commonly considered important for an anti-tumor effect. We recently showed that antibodies generated by dogs with cancer could be detected on an 800 FS peptide array. Peptides reactive on the dog array, whose homolog was also present in a mouse tumor cell line, were protective in the mouse models, while non-reactive peptides on the array did not confer protection. This study establishes that antibody response is an indicator of vaccine effectiveness. We also demonstrated in that work that the level of antibody response correlated with protection in the mouse models. One explanation for this observation is that the IgG antibody response depends on CD4+ T-cell help. FS with good CD4+ T cell epitopes may also elicit tumor cell killing. It has been noted that CD4+ T cell responses to vaccines correlate with protection^66,67^.

In summary, we have discovered another class of neoantigens that may be useful in developing different types of cancer vaccines. We have also created an array format for directly detecting immune responses to these tumor antigens. It will be important to determine if these antigens and the method to detect them have clinical utility. We strongly believe that the data presented, as well as more to be submitted, support bringing FS antigen cancer vaccines to clinical trial. We have recently initiated a large dog clinical trial of a pan-cancer prophylactic vaccine and will soon submit protocols for both dog and human therapeutic trials of cancer-type specific vaccines.

## Supplementary information

Supplemental Figures and Tables

Table S2

Table S1
