# Author Correction: RNA Transcription and Splicing Errors as a Source of Cancer Frameshift Neoantigens for Vaccines

**DOI:** 10.1038/s41598-019-54300-0

**Published:** 2019-11-25

**Authors:** Luhui Shen, Jian Zhang, HoJoon Lee, Milene Tavares Batista, Stephen Albert Johnston

**Affiliations:** 1 The Biodesign Institute Center for Innovations in Medicine, Arizona State University, Tempe, AZ USA; 2 Present Address: Stanford University, Stanford, CA USA

Correction to: *Scientific Reports* 10.1038/s41598-019-50738-4, published online 02 October 2019

The original version of this Article contained a typographical error in the spelling of the author HoJoon Lee, which was incorrectly given as Hojoon Lee. This has now been corrected in the PDF and HTML versions of the Article, and in the accompanying Supplementary Information file.

