# Author Correction: RNA Transcription and Splicing Errors as a Source of Cancer Frameshift Neoantigens for Vaccines

**DOI:** 10.1038/s41598-020-63114-4

**Published:** 2020-04-07

**Authors:** Luhui Shen, Jian Zhang, HoJoon Lee, Milene Tavares Batista, Stephen Albert Johnston

**Affiliations:** 1 The Biodesign Institute Center for Innovations in Medicine, Arizona State University, Tempe, AZ USA; 2 Present Address: Stanford University, Stanford, CA USA

Correction to: *Scientific Reports* 10.1038/s41598-019-50738-4, published online 02 October 2019

This Article contains a typographical error in the Supplementary information section where,

“Table S2”

should read:

“Table S3”

