# Supplementary material for: RNA Transcription and Splicing Errors as a Source of Cancer Frameshift Neoantigens for Vaccines
Source: Sci Rep. 2019 Oct 2;9:14184. doi: 10.1038/s41598-019-50738-4 (PMC6775166; doi:10.1038/s41598-019-50738-4)
Supplement: Supplementary file 1 — Supplemental Figures and Tables [file 41598_2019_50738_MOESM1_ESM.pdf]

RNA Transcription and Splicing Errors as a Source of Cancer Frameshift Neoantigens for Vaccines

Authors:
Luhui Shen[1,3], Jian Zhang[1,3], HoJoon Lee[1,2], Milene Tavares Batista[1], Stephen Albert Johnston[1,*]

Affiliations:
1.The Biodesign Institute Center for Innovations in Medicine, Arizona State University, Tempe, AZ
2.H.L present address: Stanford University, Stanford, CA
3.These authors contributed equally

L.S. and J.Z. are co-first authors of this article.

*Correspondence: Stephen Albert Johnston, Stephen.Johnston@asu.edu

Supplemental Figures and Tables:

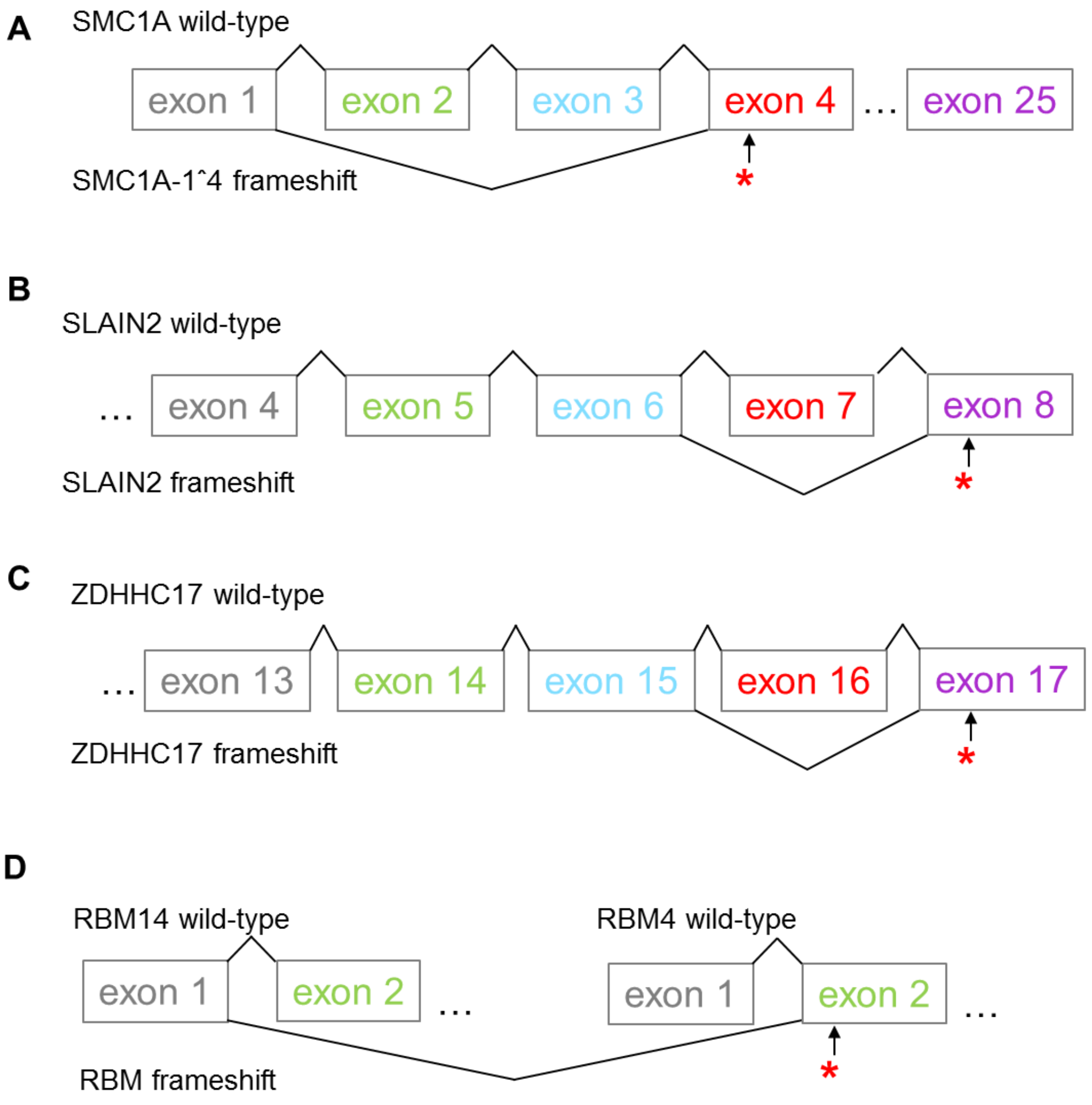
A
SMC1A wild-type
exon 1
exon 2
exon 3
exon 4
…
exon 25
SMC1A-1ˆ4 frameshift
*
B
SLAIN2 wild-type
…
exon 4
exon 5
exon 6
exon 7
exon 8
SLAIN2 frameshift
*
C
ZDHHC17 wild-type
…
exon 13
exon 14
exon 15
exon 16
exon 17
ZDHHC17 frameshift
*
D
RBM14 wild-type
exon 1
exon 2
…
RBM4 wild-type
exon 1
exon 2
…
RBM frameshift
*

E

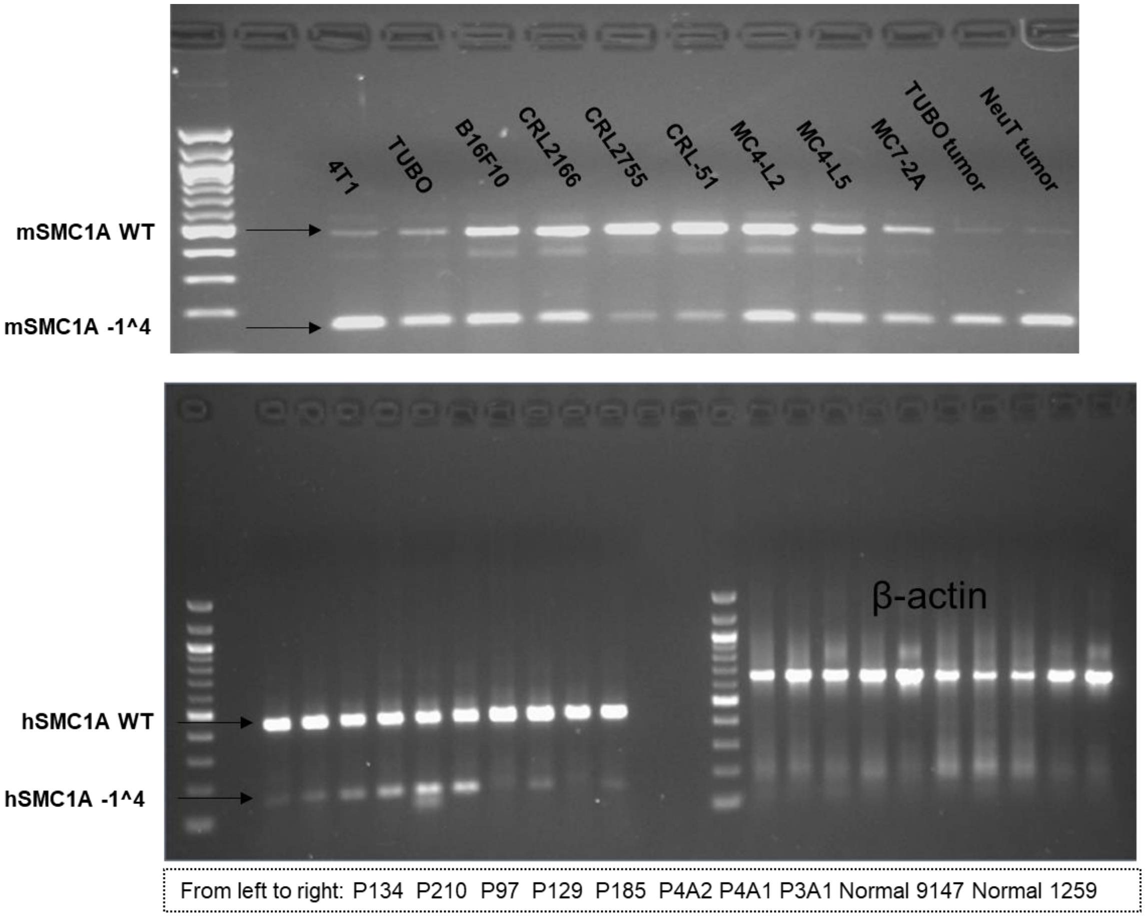
4T1
TUBO
B16F10
CRL2166
CRL2755
CRL-51
MC4-L2
MC4-L5
MC7-2A
TUBO tumor
NeuT tumor
mSMC1A WT
mSMC1A -1^4
β-actin
hSMC1A WT
hSMC1A -1^4
From left to right: P134 P210 P97 P129 P185 P4A2 P4A1 P3A1 Normal 9147 Normal 1259

F.

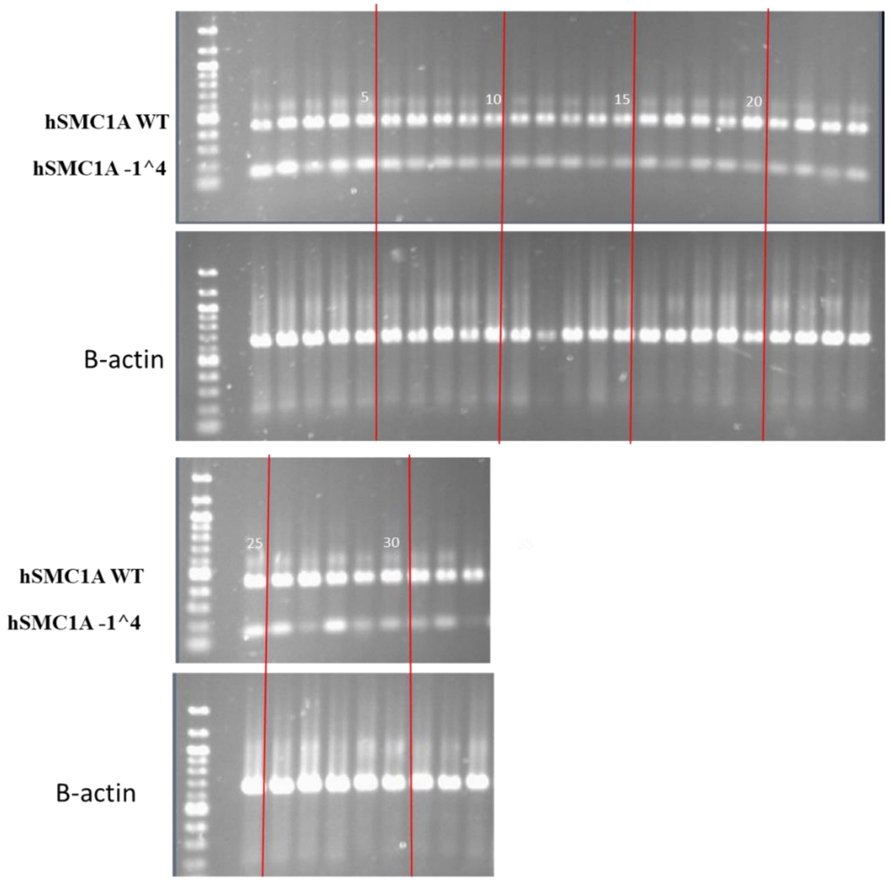

1	MDA-MB-468
2	SW 527
3	HS 606T
4	HCC1187
5	HCC1806
6	HCC1395
7	MDA-MB-453
8	HCC 1143
9	M4A4
10	HCC1419
11	HCC70
12	UACC-893
13	AU565
14	HCC 1954
15	ZR-75-30
16	HCC1937
17	HCC1428
18	MCF-7
19	Hs-578.T
20	HCC1500
21	MB157
22	Hs739.T
23	DU4475
24	MDA-MB-231
25	Cama-1
26	ZR-75-1
27	HS-362.T
28	BT-20
29	HCC 2218
30	HCC 1599
31	Hs 605.T
32	BT474
33	Hs 343.T

G.

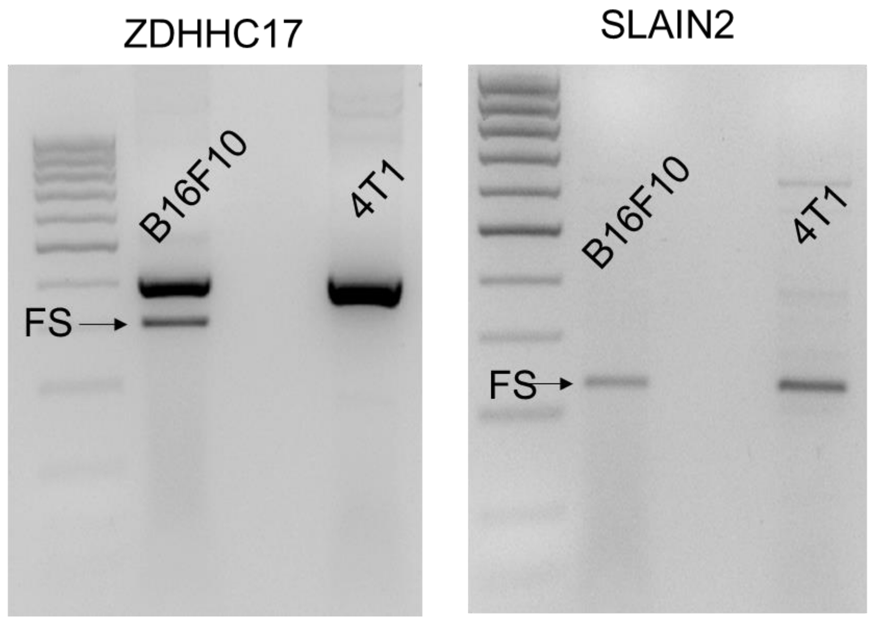

H.

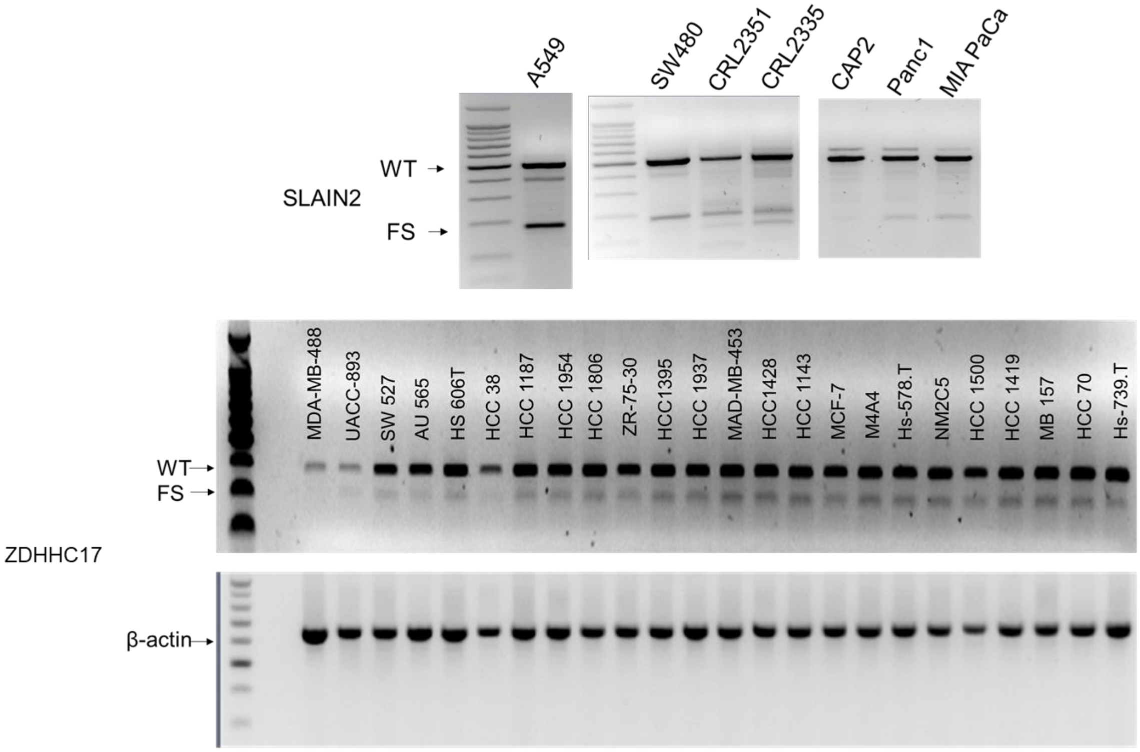
A549
SW480
CRL2351
CRL2335
CAP2
Panc1
MIA PaCa
WT
SLAIN2
FS
MDA-MB-488
UACC-893
SW 527
AU 565
HS 606T
HCC 38
HCC 1187
HCC 1954
HCC 1806
ZR-75-30
HCC1395
HCC 1937
MAD-MB-453
HCC1428
HCC 1143
MCF-7
M4A4
Hs-578.T
NM2C5
HCC 1500
HCC 1419
MB 157
HCC 70
Hs-739.T
WT
FS
ZDHHC17
β-actin

I.

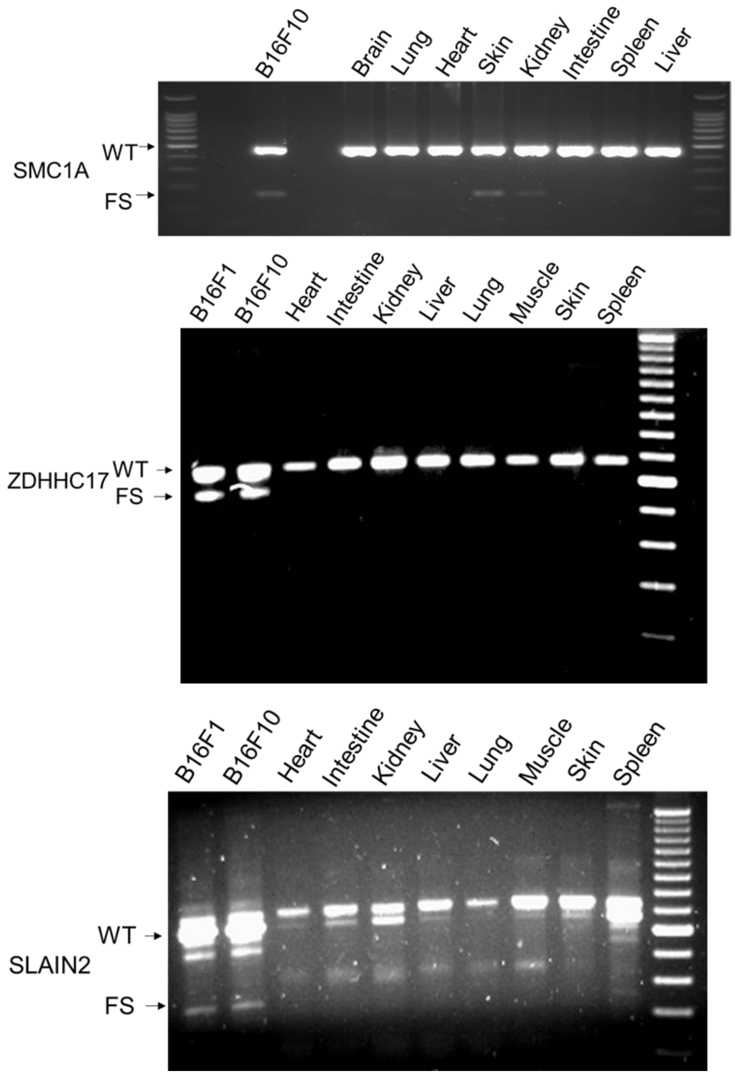

Fig. S1. Schematic of FS mis-splicing. A. Schematic of exon mis-splicing of mSMC1A. The asterisk indicates the stop codon that is generated by a shift in reading frame upon joining exon 1 with exon 4. **B.** Schematic of exon mis-splicing of ZDHHC17. The asterisk indicates the stop codon that is generated by a shift in reading frame upon joining exon 15 with exon 17. **C.** Schematic of exon mis-splicing of SLAIN2 by splicing exon 6 with exon 8. **D.** Schematic of exon mis-splicing of RBM by splicing RBM14 exon 1 with RBM4 exon 2. **E.** Full figure of Figure 2A. **F.** RT-PCR of human SMC1A (hSMC1A), human SMC1A FS (hSMC1A-1^4) and β-actin in 33 human breast tumor cell lines. **G.** RT-PCR analysis of the ZDHHC17_FS and SLAIN2_FS in B16F10 and 4T1 tumor cell cDNA. **H.** RT-PCR analysis of SLAIN2_FS and ZDHHC17_FS in different human tumor cells. **I.** RT-PCR analysis of SMC1A_FS, SLAIN_FS and ZDHHC17_FS variants in B16 melanoma cells and normal tissues from C57BL6 mouse.

A

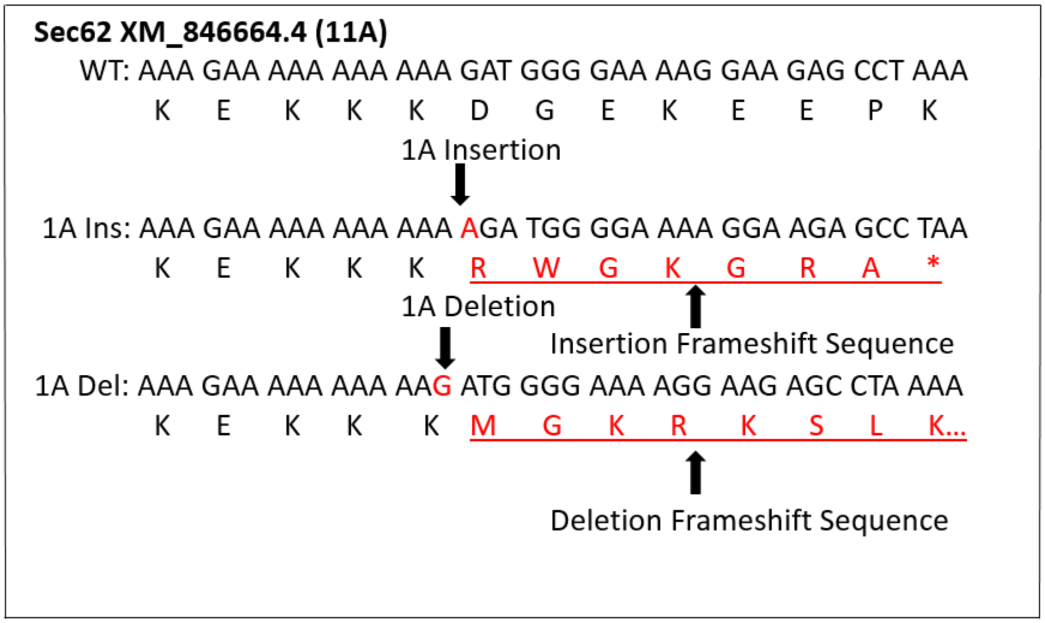

B

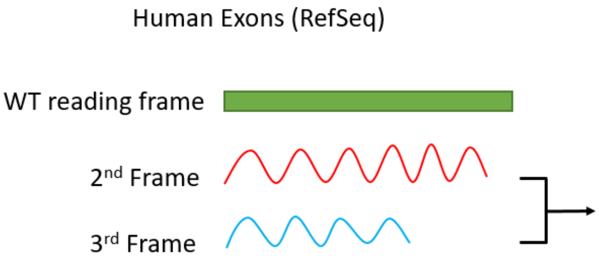

Human Exon Junction Frameshift Database

ID	Gene	Frameshift Peptide
NM_001204516.1_Exon1_2nd	KIFAP3	CKGRTPDTSK
NM_001243136.1_Exon2_2nd	PELI3	CTTSPRRSSP
NM_001301138.1_Exon1_2nd	VPS39	CTTLSSQCRS
NM_001302453.1_Exon1_2nd	CCDC151	CTLRLPPVRN
NM_001304441.1_Exon2_2nd	MMP8	CNKYLKRSQL
NM_001308192.1_Exon2_2nd	CPEB4	CTHWRVHSLT
NM_001308351.1_Exon4_2nd	ZNF433	CKKPSGTWPL
NM_014350.3_Exon1_2nd	TNFAIP8	CTPKQKNPRK
NM_139320.1_Exon3_2nd	CHRFAM7A	CKNIASTSIF

C

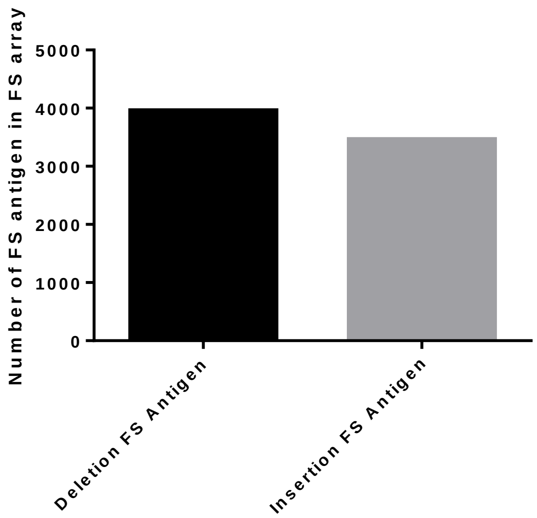
Number of FS antigen in FS array
5000
4000
3000
2000
1000
0
Deletion FS Antigen
Insertion FS Antigen

D

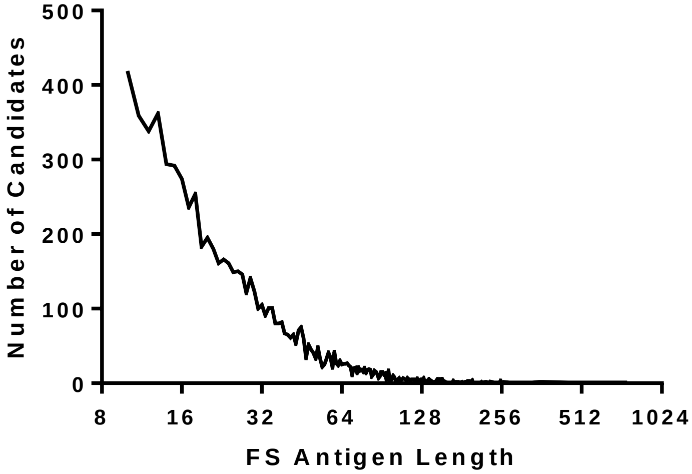
Number of Candidates
500
400
300
200
100
0
8
16
32
64
128
256
512
1024
FS Antigen Length

E

F

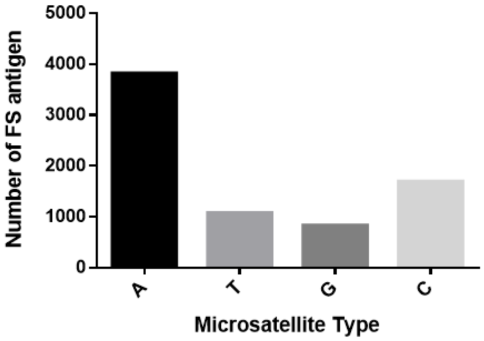
Number of FS antigen
5000
4000
3000
2000
1000
0
A
T
G
C
Microsatellite Type

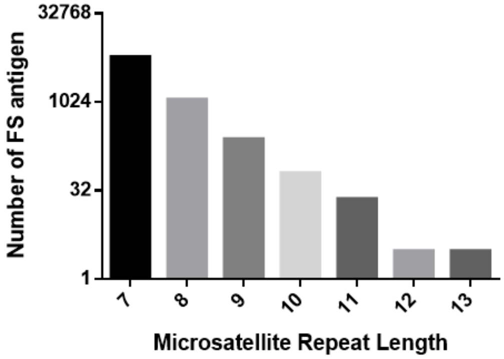
Number of FS antigen
32768
1024
32
1
7
8
9
10
11
12
13
Microsatellite Repeat Length

G

H

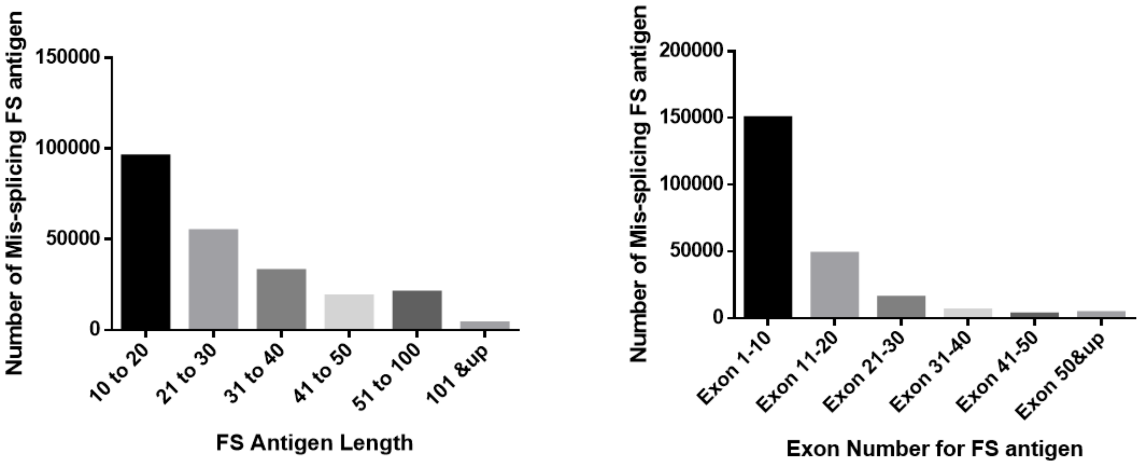

Figure S2. Components of frameshift peptide array and characteristics.

A. Example of INDEL Frameshift peptides from dog gene SEC62

B. Examples of mis-splicing Frameshift peptides from 2nd frame and 3rd frame of human exons.

C. Distribution of MS FS peptides in human FS array with insertion or deletion events

D. Distribution of MS FS peptide lengths in human FS array with corresponding FS antigen length

E. Distribution of MS Type in human FS array

F. Distribution of MS repeat length in human FS array

G. Distribution of Mis-splicing FS antigen length in human FS array

H. Distribution of Exon numbers of FS antigens in human FS array

A

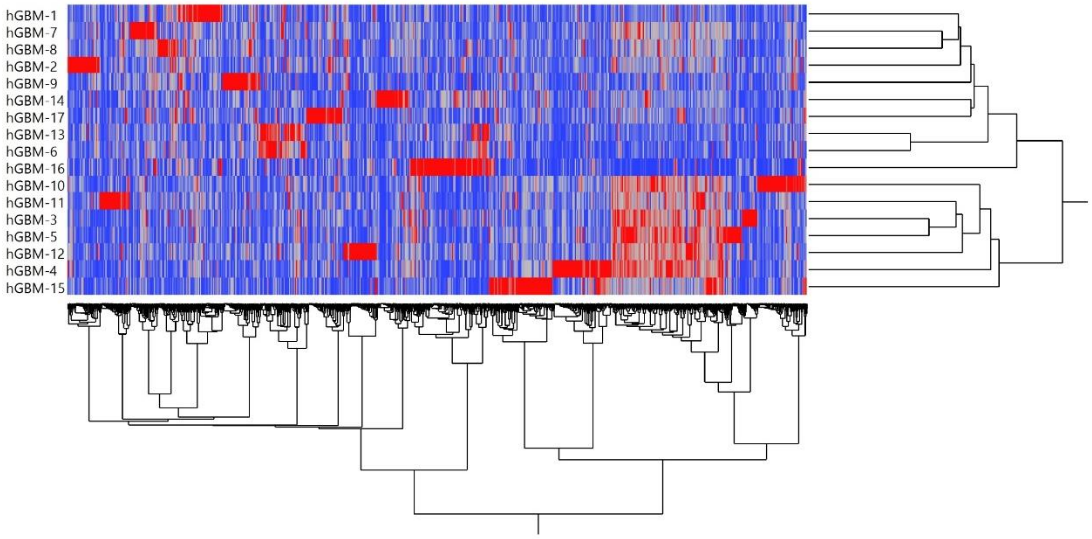

B

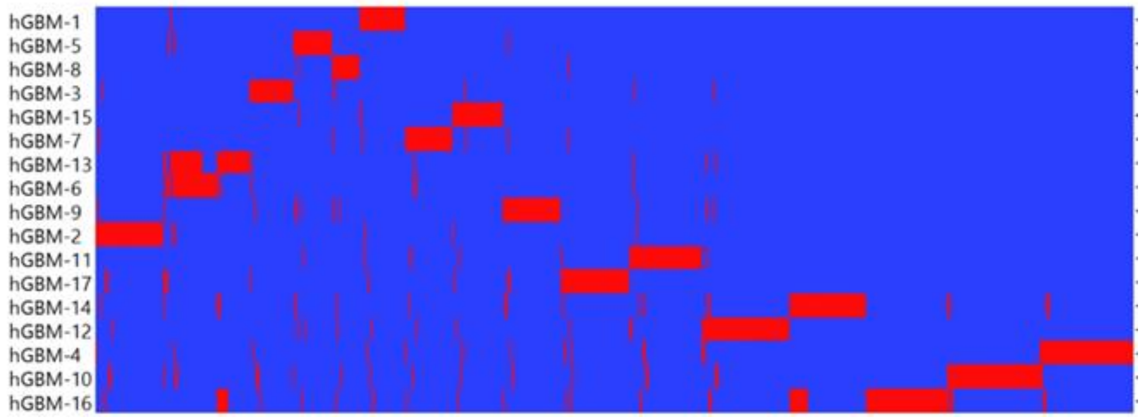
hGBM-1
hGBM-5
hGBM-8
hGBM-3
hGBM-15
hGBM-7
hGBM-13
hGBM-6
hGBM-9
hGBM-2
hGBM-11
hGBM-17
hGBM-14
hGBM-12
hGBM-4
hGBM-10
hGBM-16

C

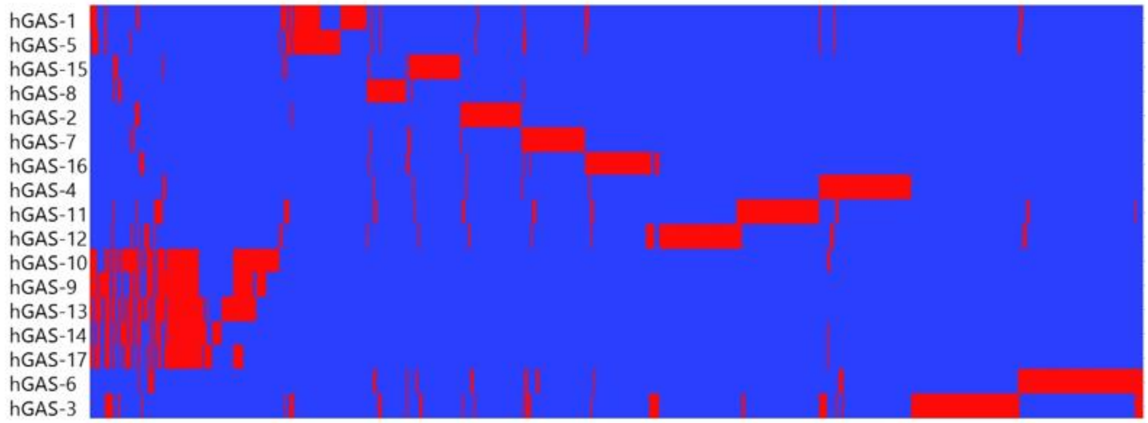
hGAS-1
hGAS-5
hGAS-15
hGAS-8
hGAS-2
hGAS-7
hGAS-16
hGAS-4
hGAS-11
hGAS-12
hGAS-10
hGAS-9
hGAS-13
hGAS-14
hGAS-17
hGAS-6
hGAS-3

D

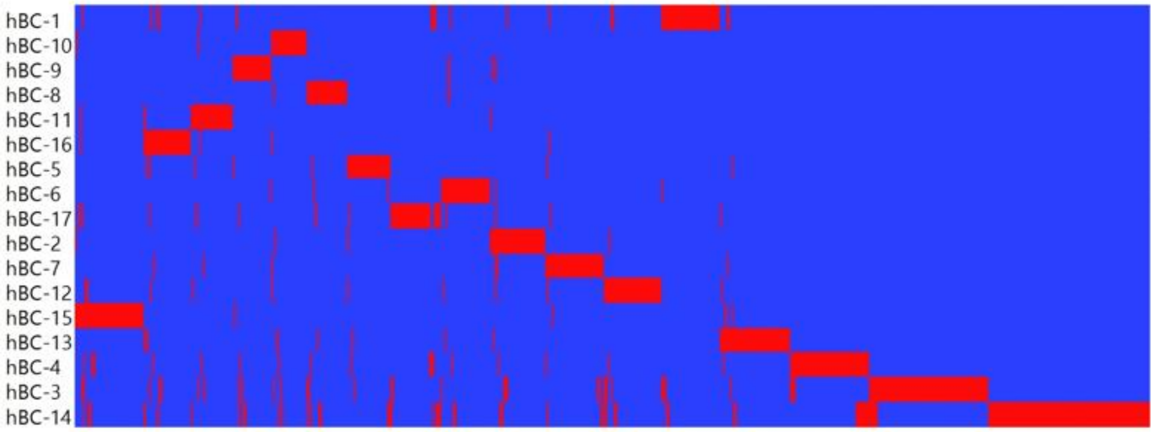
hBC-1
hBC-10
hBC-9
hBC-8
hBC-11
hBC-16
hBC-5
hBC-6
hBC-17
hBC-2
hBC-7
hBC-12
hBC-15
hBC-13
hBC-4
hBC-3
hBC-14

E

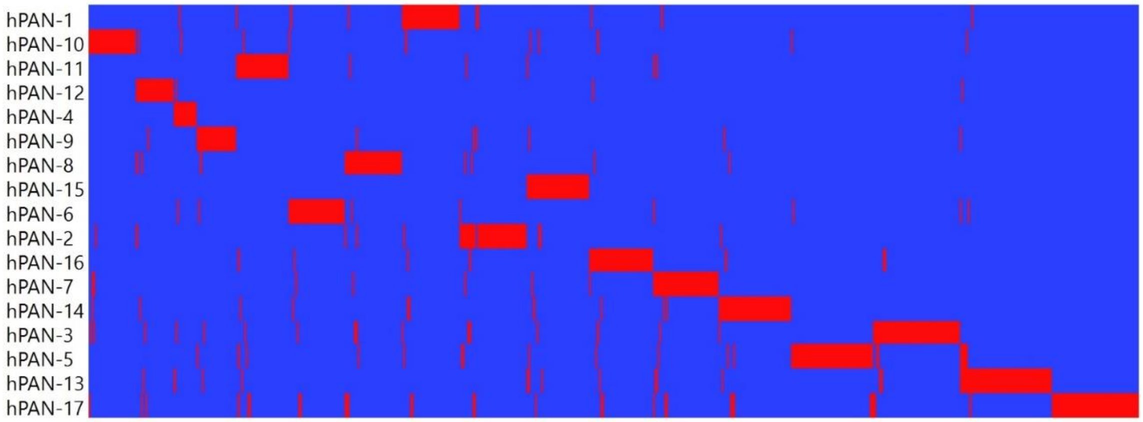
hPAN-1
hPAN-10
hPAN-11
hPAN-12
hPAN-4
hPAN-9
hPAN-8
hPAN-15
hPAN-6
hPAN-2
hPAN-16
hPAN-7
hPAN-14
hPAN-3
hPAN-5
hPAN-13
hPAN-17

F

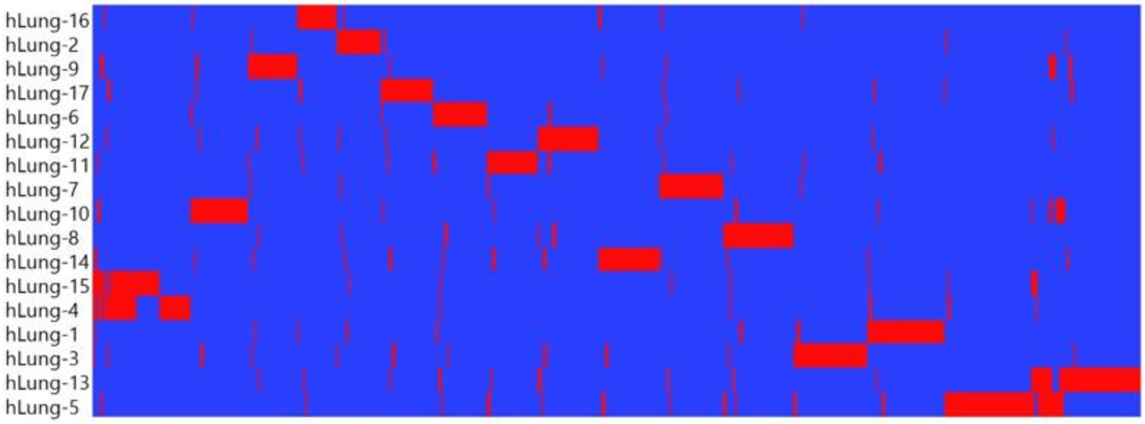
hLung-16
hLung-2
hLung-9
hLung-17
hLung-6
hLung-12
hLung-11
hLung-7
hLung-10
hLung-8
hLung-14
hLung-15
hLung-4
hLung-1
hLung-3
hLung-13
hLung-5

G

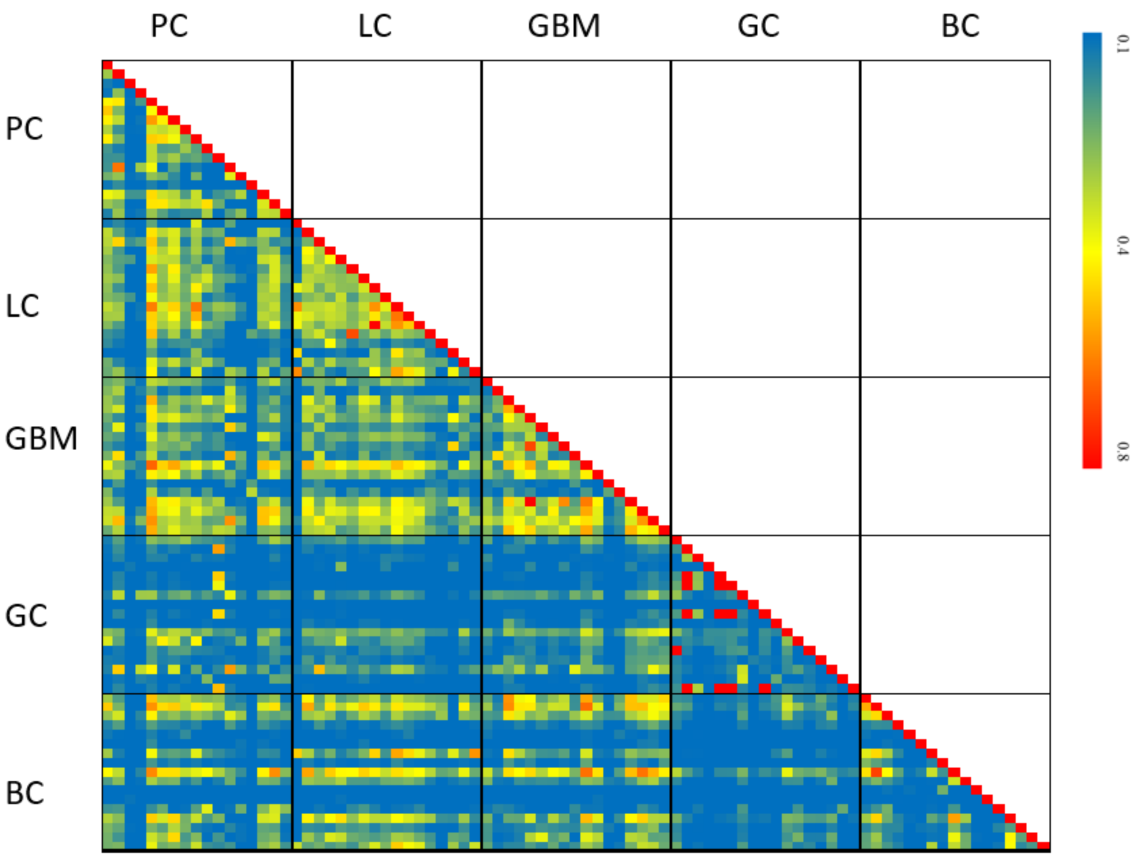

Figure S3. Personal frameshift response in 4 cancer types

A. Hierarchical clustering of all 400K FS peptides in 17 GBM samples.
B. Personal anti-FS response in 17 GBM cancer patients.
C. Personal anti-FS response in 17 gastric cancer patients.
D. Personal anti-FS response in 17 breast cancer patients.
E. Personal anti-FS response in 17 pancreatic cancer patients.
F. Personal anti-FS response in 17 lung cancer patients.
G. Correlation matrix of anti-FS response in all cancer samples from 5 cancer types

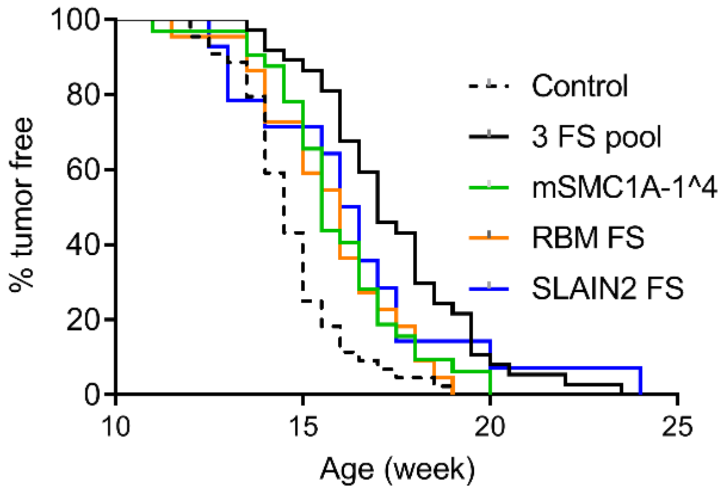

Fig S4 Tumor free curve of each FS neo-antigen immunized group in BALB-NeuN mice. BALB-NeuT mice were immunized with individual FS antigens (mSMC1A-1^4, n=32; RBM, n=22; and SLAIN2, n=14) (total n=68), pool of these three FS antigens (n=37) and control group (total n=44), including untreated (n=14) and immunized with control antigens (n=30). All of the mice were immunized with the same regime as in Fig 4D. Detail immunization regime see the method. Control v.s. each of individual FS group, $p<0.05$; 3FS pool vs. mSMC1A-1^4 or RBM FS, $p \leq 0.005$; 3FS pool vs. SLAIN2, $p=0.43$. All statistical analysis were with Mantal-Cox test. Detail immunization regimes were described in the methods.

Table S2. The 50 human breast cancer cell lines.

No.	Cell Line	ATCC_Name	Tissue
1	MCF-10A	CRL-10317	breast
2	BT-474	HTB-20	breast
3	Hs 319.T	CRL-7236	breast
4	HCC1428	CRL-2327	breast
5	HCC1599	CRL-2331	breast
6	Hs 605.T	CRL-7365	breast
7	Hs 362.T	CRL-7253	breast
8	ZR-75-1	CRL-1500	breast
9	MCF-7	HTB-22	breast
10	Hs 281.T	CRL-7227	breast
11	HCC1500	CRL-2329	breast
12	BT-20	HTB-19	breast
13	HCC1143	CRL-2321	breast
14	UACC-812	CRL-1897	breast

15	SW527	CRL-7940	breast
16	MDA-MB-453	HTB-131	breast
17	ZR-75-30	CRL-1504	breast
18	MDA-MB-468	HTB-132	breast
19	HCC1187	CRL-2322	breast
20	SK-BR-3	HTB-30	breast
21	MDA-MB-175-VII	HTB-25	breast
22	Hs 574.T	CRL-7345	breast
23	HCC 1008	CRL-2320	breast
24	Hs 742.T	CRL-7482	breast
25	Hs 748.T	CRL-7486	breast
26	BT-483	HTB-121	breast
27	HCC202	CRL-2316	breast
28	HCC 2157	CRL-2340	breast
29	BT-549	HTB-122	breast
30	MDA-MB-415	HTB-128	breast
31	HCC1395	CRL-2324	breast
32		HTB-127	breast
33	MDA-MB-231	HTB-26	breast
34	CAMA-1	HTB-21	breast
35	MDA-MB-134-VI	HTB-23	breast
36	Hs 606.T	CRL-7368	breast
37	HCC1806	CRL-2335	breast
38	HCC1419	CRL-2326	breast
39	AU565	CRL-2351	breast
40	HCC1937	CRL-2336	breast
41	Hs 578T	HTB-126	breast
42	Hs 739.T	CRL-7477	breast
43	DU4475	HTB-123	breast
44	HCC70	CRL-2315	breast
45	HCC38	CRL-2314	breast
46	HCC1954	CRL-2338	breast
47	MB 157	CRL-7721	breast
48	HCC2218	CRL-2343	breast
49	Hs 343.T	CRL-7245	breast
50	UACC-893	CRL-1902	breast

Table S4. **Mouse mis-splicing FS antigens in the vaccine**

Antigen Name	Peptide size	peptide sequence (Black: upstream wild type peptide; Red: FS peptide)
ZDHHC17 FS	21	AVLLMCQLYQPWMCKEYYRLL
SLAIN2 FS	21	IPRMQPQASANHCQLLKVMVA
mSMC1A-1^4	27	TAIIGPNGSGCSGVYCHEEPQGEDSSV
RBM FS	45	GRVIECDVVKGSCQDGEAVHWKSAPGGHRAGDPLTLRAVREGAG M

Table S5. Three mouse MS FS antigens with predicted H2-D epitope

Antige n ID	Access #	MS type	IND EL	Peptide size	peptide sequence (Kd/Ld epitope score>20)
MS927	NM_053 009.3	9_A	Del	33	ICM**SPPLLWATL**QAPETTSAACKA**SYRPEGLYL**
MS255	NM_010 086.4	9_A	In	24	YFSCDKRCIK**HYAGNKSLL**TFSGY
MS518	NM_153 511.3	10_A	Del	59	TLCMEVMLRWNTREL**GYLYLQLCFL**NTHFLHTSQEE**KL LTLGRFL**TWTSRCGSFVIRPL

Table S6. Samples tested on Human 400K FS array

Sample Type	Number of Samples	Source
Breast Cancer	17	UT Southwestern
Lung Cancer	17	UT Southwestern
GBM	17	Barrows Neurological Institute
Pancreatic Cancer	17	TGEN
Pancreatic Cancer Stage 1	13	TGEN
Gastric Cancer	17	Japan
Control	64	Varied Sources

Table S7. Three ORFs of Sec62 gene

Sec62-12A:<br>ATGGCGGAGCGCAGGAGACACAAGAAGCGGATCCAGGAAGTTGGTGAACC

ATCTAAAGAAGAGAAGGCTGTAGCCAAGTATCTTCGATTTAACTGTCCAAC<br>AAAGTCTACCAATATGATGGGGCACCGAGTTGATTATTTCATTGCTTCAAA<br>AGCAGTGGATTGCCTTTTGGATTCAAAGTGGGCAAAGGCCAAGAAAGGAG<br>AGGAAGCTTTATTTACAACAAGGGAGTCTGTGGTTGACTACTGCAACAGGC<br>TTTTAAAGAAGCAGTTTTTTCACCGGGCACTAAAAGTAATGAAAATGAAGT<br>ATGATAAAGACATAAAAAAAGAAAAAGAGAAAGGAAAGGCCGAAAGTGG<br>AAAAGAAGAAGATAAAAAGAGCAGGAAAGAAAATCTAAAGGATGAAAAG<br>ACGAAAAAGGAGAAAGAAAAAAAAAAAAGATGGGGAAAAGGAAGAGGA<br>TTACAAGGACGACGACGACAAGTGAAATTCATGGTGAGCAAGGGCGAGGA<br>GCTGTTCACCGGGGTGGTGCCCATCCTGGTCGAGCTGGACGGCGACGTAAA<br>CGGCCACAAGTTCAGCGTGTCCGGCGAGGGCGAGGGCGATGCCACCTACG<br>GCAAGCTGACCCTGAAGTTCATCTGCACCACCGGCAAGCTGCCCGTGCCCT<br>GGCCCACCCTCGTGACCACCCTGACCTACGGCGTGCAGTGCTTCAGCCACT<br>ACCCCGACCACATGAAGCAGCACGACTTCTTCAAGTCCGCCATGCCCGAAG<br>GCTACGTCCAGGAGCGCACCATCTTCTTCAAGGACGACGGCAACTACAAGA<br>CCCGCGCCGAGGTGAAGTTCGAGGGCGACACCCTGGTGAACCGCATCGAG<br>CTGAAGGGCATCGACTTCAAGGAGGACGGCAACATCCTGGGGCACAAGCT<br>GGAGTACAACTACAACAGCCACAACGTCTATATCATGGCCGACAAGCAGA<br>AGAACGGCATCAAGGTGAACTTCAAGATCCGCCACAACATCGAGGACGGC<br>AGCGTGCAGCTCGCCGACCACTACCAGCAGAACACCCCCATCGGCGACGG<br>CCCCGTGCTGCTGCCCGACAACCACTACCTGAGCACCCAGTCCGCCCTGAG<br>CAAAGACCCCAACGAGAAGCGCGATCACATGGTCCTGCTGGAGTTCGTGA<br>CCGCCGCCGGGATCACTCTCGGCATGGACGAGCTGTACAAGAGATCTGGTA<br>CCACGCGTATCGATAAGCTTGCATGCCTGCAGGTCGACTCTAGAGGATCGT<br>GA;
Sec62-11A:<br>ATGGCGGAGCGCAGGAGACACAAGAAGCGGATCCAGGAAGTTGGTGAACC<br>ATCTAAAGAAGAGAAGGCTGTAGCCAAGTATCTTCGATTTAACTGTCCAAC<br>AAAGTCTACCAATATGATGGGGCACCGAGTTGATTATTTCATTGCTTCAAA<br>AGCAGTGGATTGCCTTTTGGATTCAAAGTGGGCAAAGGCCAAGAAAGGAG<br>AGGAAGCTTTATTTACAACAAGGGAGTCTGTGGTTGACTACTGCAACAGGC<br>TTTTAAAGAAGCAGTTTTTTCACCGGGCACTAAAAGTAATGAAAATGAAGT<br>ATGATAAAGACATAAAAAAAGAAAAAGAGAAAGGAAAGGCCGAAAGTGG<br>AAAAGAAGAAGATAAAAAGAGCAGGAAAGAAAATCTAAAGGATGAAAAG<br>ACGAAAAAGGAGAAAGAAAAAAAAAAAAGATGGGGAAAAGGAAGAGGA<br>TTACAAGGACGACGACGACAAGTGAAATTCATGGTGAGCAAGGGCGAGGA<br>GCTGTTCACCGGGGTGGTGCCCATCCTGGTCGAGCTGGACGGCGACGTAAA<br>CGGCCACAAGTTCAGCGTGTCCGGCGAGGGCGAGGGCGATGCCACCTACG<br>GCAAGCTGACCCTGAAGTTCATCTGCACCACCGGCAAGCTGCCCGTGCCCT<br>GGCCCACCCTCGTGACCACCCTGACCTACGGCGTGCAGTGCTTCAGCCACT<br>ACCCCGACCACATGAAGCAGCACGACTTCTTCAAGTCCGCCATGCCCGAAG<br>GCTACGTCCAGGAGCGCACCATCTTCTTCAAGGACGACGGCAACTACAAGA<br>CCCGCGCCGAGGTGAAGTTCGAGGGCGACACCCTGGTGAACCGCATCGAG<br>CTGAAGGGCATCGACTTCAAGGAGGACGGCAACATCCTGGGGCACAAGCT<br>GGAGTACAACTACAACAGCCACAACGTCTATATCATGGCCGACAAGCAGA<br>AGAACGGCATCAAGGTGAACTTCAAGATCCGCCACAACATCGAGGACGGC

AGCGTGCAGCTCGCCGACCACTACCAGCAGAACACCCCCATCGGCGACGG<br>CCCCGTGCTGCTGCCCGACAACCACTACCTGAGCACCCAGTCCGCCCTGAG<br>CAAAGACCCCAACGAGAAGCGCGATCACATGGTCCTGCTGGAGTTCGTGA<br>CCGCCGCCGGGATCACTCTCGGCATGGACGAGCTGTACAAGAGATCTGGTA<br>CCACGCGTATCGATAAGCTTGCATGCCTGCAGGTCGACTCTAGAGGATCGT<br>GA;
Sec62-Non MS:<br>ATGGCGGAGCGCAGGAGACACAAGAAGCGGATCCAGGAAGTTGGTGAACC<br>ATCTAAAGAAGAGAAGGCTGTAGCCAAGTATCTTCGATTTAACTGTCCAAC<br>AAAGTCTACCAATATGATGGGGCACCGAGTTGATTATTTCATTGCTTCAAA<br>AGCAGTGGATTGCCTTTTGGATTCAAAGTGGGCAAAGGCCAAGAAAGGAG<br>AGGAAGCTTTATTTACAACAAGGGAGTCTGTGGTTGACTACTGCAACAGGC<br>TTTTAAAGAAGCAGTTTTTTCACCGGGCACTAAAAGTAATGAAAATGAAGT<br>ATGATAAAGACATAAAAAAAGAAAAAGAGAAAGGAAAGGCCGAAAGTGG<br>AAAAGAAGAAGATAAAAAGAGCAGGAAAGAAAATCTAAAGGATGAAAAG<br>ACGAAAAAGGAGAAAGAGAGGAAGAGAGATGGGGAAAAGGAAGAGGATT<br>ACAAGGACGACGACGACAAGTGAAATTCATGGTGAGCAAGGGCGAGGAGC<br>TGTTCACCGGGGTGGTGCCCATCCTGGTCGAGCTGGACGGCGACGTAAACG<br>GCCACAAGTTCAGCGTGTCCGGCGAGGGCGAGGGCGATGCCACCTACGGC<br>AAGCTGACCCTGAAGTTCATCTGCACCACCGGCAAGCTGCCCGTGCCCTGG<br>CCCACCCTCGTGACCACCCTGACCTACGGCGTGCAGTGCTTCAGCCACTAC<br>CCCGACCACATGAAGCAGCACGACTTCTTCAAGTCCGCCATGCCCGAAGGC<br>TACGTCCAGGAGCGCACCATCTTCTTCAAGGACGACGGCAACTACAAGACC<br>CGCGCCGAGGTGAAGTTCGAGGGCGACACCCTGGTGAACCGCATCGAGCT<br>GAAGGGCATCGACTTCAAGGAGGACGGCAACATCCTGGGGCACAAGCTGG<br>AGTACAACTACAACAGCCACAACGTCTATATCATGGCCGACAAGCAGAAG<br>AACGGCATCAAGGTGAACTTCAAGATCCGCCACAACATCGAGGACGGCAG<br>CGTGCAGCTCGCCGACCACTACCAGCAGAACACCCCCATCGGCGACGGCC<br>CCGTGCTGCTGCCCGACAACCACTACCTGAGCACCCAGTCCGCCCTGAGCA<br>AAGACCCCAACGAGAAGCGCGATCACATGGTCCTGCTGGAGTTCGTGACC<br>GCCGCCGGGATCACTCTCGGCATGGACGAGCTGTACAAGAGATCTGGTACC<br>ACGCGTATCGATAAGCTTGCATGCCTGCAGGTCGACTCTAGAGGATCGTGA<br>.
